# Improvement of CRISPR/Cas9 system by transfecting Cas9-expressing *Plasmodium berghei* with linear donor template

**DOI:** 10.1038/s42003-020-01138-2

**Published:** 2020-08-05

**Authors:** Naoaki Shinzawa, Tsubasa Nishi, Fumiya Hiyoshi, Daisuke Motooka, Masao Yuda, Shiroh Iwanaga

**Affiliations:** 1 Department of Environmental Parasitology, Graduate School of Medical and Dental Sciences, Tokyo Medical and Dental University, 1-5-45 Yushima, Bunkyo-ku, Tokyo, 113-8519 Japan; 2 Laboratory of Medical Zoology, Department of Medicine, Mie University, Tsu, 514-8507 Mie Japan; 3 Department of Infection Metagenomics, Genome Information Research Center, Research Institute for Microbial Diseases, Osaka University, Suita, 565-0871 Osaka Japan; 4 Department of Molecular Protozoology, Research Institute for Microbial Diseases, Osaka University, Suita, 565-0871 Osaka Japan

**Keywords:** CRISPR-Cas9 genome editing, Malaria

## Abstract

Malaria is caused by infection with *Plasmodium* parasites and is a major public health concern. The CRISPR/Cas9 system is a promising technology, but still has technical problems, such as low efficiency and unexpected recombination. Here, we solved these problems by transfecting Cas9-expressing parasites with linear donor templates. The use of a linear donor template prevented unexpected recombination; in addition, constitutive expression of Cas9 enabled immediate cleavage of the target locus after transfection, allowing efficient integration of the donor template. Furthermore, due to the absence of the cNHEJ pathway, there were no off-target mutations in the resultant parasites. In addition, this developed method could be applied for multiple genetic modifications on different chromosomes and for large-scale chromosomal deletion in the subtelomeric region. Because of its robustness, high efficiency, and versatile applicability, we hope this method will be standard in the post-genomic era of *Plasmodium* species.

## Abstract (web summary)

Shinzawa et al. develop a method that improves the transfection efficiency for *Plasmodium* parasites that cause malaria by transfecting Cas9-expressing parasites with linear donor templates. This method allows efficient integration of the donor template while preventing unexpected recombination.

## Introduction

Malaria is one of the most serious public health problems, affecting 200 million people and causing ~450,000 deaths each year. Rodent malaria parasites are safe models to study human malaria and are widely used not only for exploring drug targets and vaccine antigens but also for investigating the molecular basis of various biological events, such as host cell invasion, immune evasion, and sexual development. The genetic modification of these parasites is an essential tool for such studies and has been performed in the past by integrating the DNA fragments carrying drug-selectable marker genes into the genome by homologous recombination^1,2^. Using this method, gene functions have been analyzed by site-directed mutagenesis, gene deletion, and fusion with fluorescent proteins. Furthermore, ~2500 genes representing more than half of the protein-coding genes were systematically disrupted, and a phenotype database of individual genes was created^3^. Currently, combinations of genetic modifications are required to investigate the relationship of multiple molecules, which has improved our understanding of the precise role of each gene in the life cycle. In addition, multiple genetic modifications can be utilized to evaluate the antigenicity of multiple proteins and to investigate their synergistic effects on drug resistance. However, the number of available drug-selectable marker genes is limited, and an additional procedure is thus required to generate transgenic parasites with multiple genetic modifications; the drug-selectable marker must be recycled by first removing it from the genome using a negative selection marker^4^.

The CRISPR/Cas9 nuclease system is a powerful genome editing technique employed in various living organisms. This system can modify the targeted genes more efficiently than previous methods based on homologous recombination, and it has been previously applied for the genetic modification of *Plasmodium* species, such as *Plasmodium falciparum* and *P. yoelii*, resulting in the generation of marker-free transgenic parasites^5–11^. In principle, this system consists of the following two steps: (1) the initial cleavage of the targeted genomic locus by Cas9 and the single-guide RNA (sgRNA) complex, and (2) the genetic modification by the subsequent repair of the cleavage site. Since *Plasmodium* species lack the components of the canonical nonhomologous end-joining (cNHEJ) pathway^12^, a cleaved genomic locus is repaired either by homology-directed repair (HDR) using the donor template or by an alternative end-joining pathway, such as the microhomology-mediated end-joining pathway (MMEJ)^12–14^. However, since repair by MMEJ is infrequent^13^, HDR is typically used to repair the cleavage site in the current CRISPR/Cas9 system in parasites. For the parasites to stably maintain Cas9, the sgRNA, and the donor template, they are introduced into the parasites using two plasmids: one plasmid encoding the Cas9 gene and another plasmid encoding the sgRNA and the donor template are used^5–7^. However, because the parasites easily lose plasmids during cell division due to their low segregation efficiencies, it is difficult to obtain transgenic parasites with plasmids by drug screening, resulting in less efficient genetic modifications. In a previous study, we developed a genetic modification method using *P. falciparum* constitutively expressing Cas9 by a centromere plasmid^15^. Because the centromere plasmid segregates into daughter parasites precisely due to the function of the centromere, the parasites can express Cas9 more stably than parasites transfected with conventional plasmids. We could engineer the gene of interest with almost 100% efficiency at ~3 weeks by transfecting the Cas9-expressing *P. falciparum* with the plasmid containing both the sgRNA and the donor template. This result showed that the constitutive expression of Cas9 increased the likelihood of the coexistence of the three elements and prompted cleavage of the target locus, resulting in an efficient genetic modification.

In this study, to further improve the CRISPR/Cas9 system of the parasite, we integrated the Cas9 gene into the genome of a rodent malaria parasite, *P. berghei*, and generated a transgenic parasite that constitutively expressed Cas9. When we used the circular plasmid carrying both the donor template and the sgRNA as in the conventional CRISPR/Cas9 system, an additional copy was unexpectedly incorporated at the target genomic locus by single crossover recombination, indicating that it was a serious overlooked technical limitation of the CRISPR/Cas9 system to use plasmid DNA in the parasites. We solved this technical limitation by using a linear donor template and succeeded to engineer genes with high accuracy without any unexpected recombination. Furthermore, multiple modifications at different genomic loci and truncations of the subtelomeric region could be carried out by the developed CRISPR/Cas9 system. We further discuss the principle of genetic modification by the CRISPR/Cas9 system in parasites and the possible application of the developed system.

## Results

### Generation of *P. berghei* with an integrated Cas9 nuclease gene

**Fig. 1:**
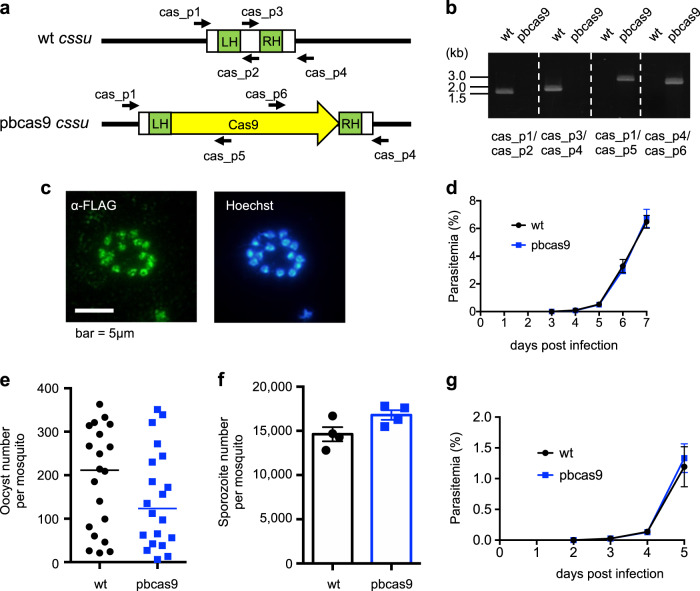
Generation and characterization of the pbcas9 parasite. **a** The Cas9 expression cassette (yellow) was integrated into the *cssu* locus by double crossover recombination using two homologous regions (green). **b** The genotyping PCR was performed using the sets of primers indicated at the bottom. The uncropped gel image of are shown in Supplementary Fig. 13. **c** The expression and localization of Cas9 was detected by anti-FLAG antibody in schizonts. **d** The growth of the pbcas9 parasites, which is indicated by the blue line, was comparable with that of the wild-type parasites *P. berghei* ANKA, which is shown by the black line. Positive and negative error was calculated from the standard error of the mean from biological triplicates. Distributions for each day were compared using the unpaired *t*-test (not significant). **e** The oocyst intensity of pbcas9 parasites in *A. stephensi* mosquitoes was comparable with that of wild-type parasites. Each dot represents oocyst numbers from individual midguts, and the horizontal lines indicate the median number of oocysts. Distributions were compared using the Mann–Whitney test (*n* = 20; not significant). **f** The sporozoite intensity of pbcas9 parasites in mosquito salivary glands was comparable with that of wild-type parasites. Five salivary glands were pooled, and the number of sporozoites present in each gland was counted. The column and error bar indicate the mean and standard error from biological quadruplicates. Distributions were compared using the unpaired *t*-test (not significant). **g** The growth of pbcas9 parasites (blue, box) in infected rats after sporozoite infection was comparable with that of wild-type parasites (black, circle). Positive and negative error was calculated from the standard error of the mean from biological quadruplicates. Distributions for each day were compared using the unpaired *t*-test (not significant).

The Cas9 nuclease gene from *Streptococcus pyogenes* was integrated into the genome of *P. berghei* using conventional methods based on homologous recombination with positive and negative drug selection markers. The DNA fragments encoding two expression cassettes of the Cas9 and *hdhfr–yfcu* genes were cloned into the plasmid (Supplementary Fig. 1a) and flanked with two partial sequences of the rRNA C-type subunit, *cssu*, which is located on chromosome 5. The transgenic parasites in which those two cassettes were incorporated were selected by positive screening with pyrimethamine, and subsequently the *hdhfr–yfcu* cassette was removed through negative selection by screening with 5-fluorocytosine (5-FC) (Supplementary Fig. 1a). We eventually cloned a parasite, in which only the Cas9 expression cassette was integrated at the *cssu* locus, by limiting dilution and named it pbcas9 (Fig. 1a). Genotyping PCR showed the correct integration of the Cas9 expression cassette at the *cssu* locus (Fig. 1b and Supplementary Fig. 13). In addition, western blot and immunofluorescence analyses showed the expression and proper nuclear localization of Cas9 (Fig. 1c and Supplementary Fig. 1b). Similar results were obtained in two biologically independent lines of pbcas9 parasites, which were generated by another transfection experiment. We further examined whether the life cycle of pbcas9 was affected by integration of the Cas9 expression cassette at the *cssu* locus and constitutive expression of Cas9. In particular, to determine the effect of long-term maintenance, we used pbcas9 parasites that were passaged over several generations. Results showed that the pbcas9 parasites were able to grow in erythrocytes with a multiplication rate comparable with that of wild-type parasites, indicating that there was no growth defect in the asexual stage (Fig. 1d and Supplementary Fig. 1c). In addition, they were able to transform into ookinetes, which is the invasion form at the mosquito stage, with a normal conversion rate in vitro (Supplementary Fig. 1d, e). Although the number of pbcas9 oocysts in the midgut was slightly lower than for wild-type parasites, they produced comparable numbers of salivary gland sporozoites (Fig. 1e, f). The prepatent period of the pbcas9 parasites was identical to that of wild type (Fig. 1g and Supplementary Fig. 1f), indicating that they could infect hepatocytes with comparable efficiency and multiply normally within those cells. Collectively, these data show that there was no obvious effect on pbcas9 development due to constitutive expression of Cas9 and integration of its expression cassette at the *cssu* locus. Previous studies had reported that disruption of the *cssu* locus caused a slight retardation in oocyst development^16^, suggesting that the slight decrease in oocyst number observed in the current study could be caused by disruption of the *cssu* locus due to integration of the cas9 expression cassette. Thus, if our CRISPR/Cas9 system should be used to analyze the function of genes involved in oocyst development, it may be better to integrate the Cas9 cassette into a different genomic locus, such as the SIL6 intergenic region^17^.

Next, to examine the effect of constitutive Cas9 expression on genomic integrity, we conducted a genome-wide sequence analysis of the pbcas9 parasites and examined whether the deleterious mutations caused by Cas9 were accumulated during maintenance. To this end, genomic DNA was purified from parasites that had been maintained over several generations. The genome was sequenced to a depth of approximately ×43.3 coverage, followed by comparison with the reference genomic sequence of *P. berghei* strain ANKA deposited in the database (PlasmoDB; https://plasmodb.org/plasmo/). We called a total of 407 mutations in pbcas9 (Supplementary Data 2), of which 177 mutations were found in not only intergenic regions, but also in A/T rich and repetitive sequences in exons and introns. Those mutations were possibly false positives because mapping errors frequently occur in those regions due to their low sequence complexity. Furthermore, 185 mutations were found in subtelomeric regions, where there are multigene families (Supplementary Data 2). Because the genomic sequence of subtelomeric regions are still incomplete, sequencing reads for multigene families were mapped incorrectly. Thus, those 185 mutations were also probably false positives. Three nonsynonymous mutations were called in the coding regions of genes (Supplementary Data 2). These mutations were also found by resequencing analysis in the parental ANKA strain of *P. berghei* used for generating the pbcas9, indicating that they were not caused by the constitutive Cas9 expression. We found 42 other mutations in this analysis (Supplementary Fig. 2 and Supplementary Data 2), but these were sequencing errors in the reference genome because they were commonly found in the recent whole-genome sequencing data of *P. berghei* ANKA (accession number: ERR3060803). Therefore, we concluded that the constitutive expression of Cas9 did not cause unexpected mutations in the parasite genome.

### Failure of genetic modification due to unexpected recombination of plasmid DNA

**Fig. 2:**
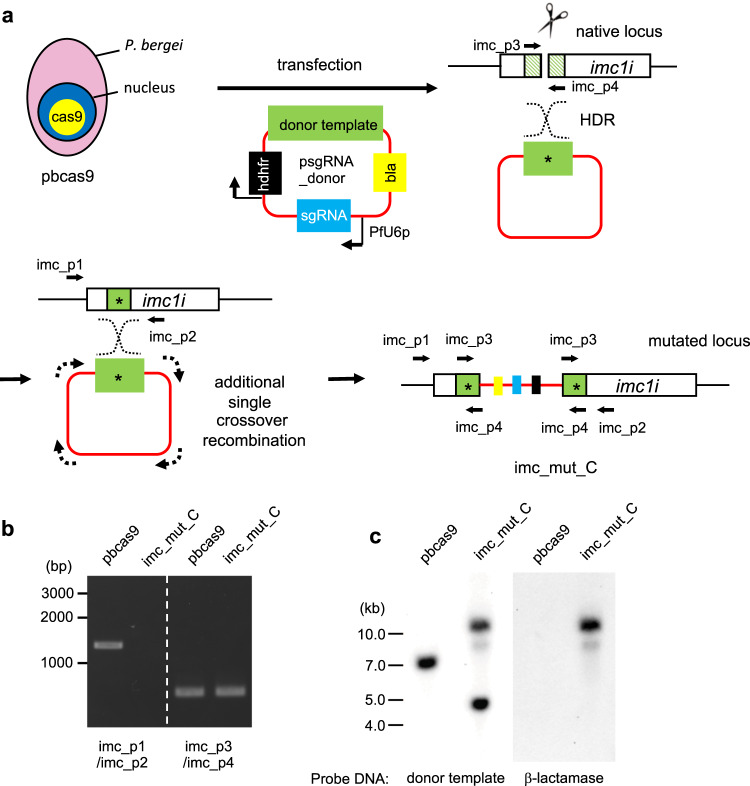
Genetic modification by the CRISPR/Cas9 system using a plasmid containing both donor template and sgRNA. **a** The pbcas9 parasites were transfected with psgRNA_donor, which contained both donor template and sgRNA. The donor template was integrated at the *imc1i* locus after cleavage of Cas9, followed by additional single crossover recombination of the psgRNA_donor plasmid at the same locus. The resulting *imc1i* locus was disrupted by the integration of the plasmid sequence. The lined boxes on native locus are the regions used for HDR. **b** The genotyping PCR of imc_mut_C was performed using the sets of primers indicated at the bottom. **c** Southern hybridization analyses of imc_mut_C were carried out using DNA fragments of the donor template and β-lactamase gene as the DNA probes. The used imc_mut_C were parasite population before cloning by limiting dilution. The uncropped images of gel or blot are shown in Supplementary Fig. 13.

To examine whether the pbcas9 parasites could be used for genetic modification, we attempted to disrupt the function of a gene by site-directed mutagenesis. The inner membrane complex 1i (*imc1i*) gene was selected as the target gene since IMC1i is a component of the glideosome of ookinetes and is dispensable for the erythrocytic developmental stage^18^. We attempted to introduce a nonsense mutation in the coding region of *imc1i* using a donor template in which we substituted the Tyr15 and Glu18 codons of the *imc1i* gene to termination codons. The donor template included not only the above nonsense mutations but also a shield mutation, i.e., mutation in the protospacer-adjacent motif (PAM), preventing recleavage by Cas9. The guide RNA was designed using the sequence of the coding region from Tyr15 to Lys21. Both the donor template and the fragment encoding the guide RNA were cloned into a circular plasmid called psgRNA_donor, which contained the *hdhfr* gene as a drug-selectable marker (Fig. 2a). The pbcas9 parasites were transfected with the resultant plasmid (5 μg) and treated with pyrimethamine for 5 days from 30 h post transfection. The parasites were visible in peripheral blood 2 days after drug withdrawal. The obtained transgenic parasite was named imc_mut_Circular, indicated as imc_mut_C. To examine whether the donor template was integrated at the *imc1i* locus of the imc_mut_C, we amplified the region targeted for HDR by PCR using the primers imc_p1 and p2, which were designed to bind outside of this region (Fig. 2a). However, no fragments were amplified by this PCR (Fig. 2b and Supplementary Fig. 13). On the other hand, when we used the primers imc_p3 and p4, which were designed to bind within the region used for HDR, the *imc1i* fragment could be amplified (Fig. 2b and Supplementary Fig. 13). The sequence analysis of the obtained fragment showed that the codons of Tyr15 and Glu18 were mutated to termination codons (Supplementary Fig. 3a). Similar results were obtained using the same primer sets in biologically independent replicates, which were obtained by another transfection experiment. These results suggested that HDR occurred between the donor template and the *imc1i* locus, while unexpected recombination might occur at the genomic locus of *imc1i* (Fig. 2a). To analyze the genomic locus of *imc1i*, we performed southern hybridization analysis of the imc_mut_C using the donor template as the DNA probe. A single signal was detected at ~7.3 kb in the wild-type parasite, but two signals were detected at 4.6 and 11.4 kb in the imc_mut_C clone (Fig. 2c and Supplementary Figs. 3b and 13). Based on the sizes of the detected signals, we presumed that the introduced plasmid might have been incorporated at the *imc1i* locus in the transgenic parasite. To confirm this, we performed southern hybridization analysis using the β-lactamase (*bla*) gene, which is present in the plasmid backbone sequence, as the DNA probe. The signal of the *bla* gene was detected at 11.4 kb, indicating the incorporation of the plasmid sequence at the *imc1i* locus (Fig. 2c and Supplementary Figs. 3b and 13). Taking all results into consideration, we concluded that the donor template was integrated once into the cleaved *imc1i* locus, and a single crossover recombination then occurred between the integrated donor template and another copy of the plasmid containing the donor template (Fig. 2a).

When the target genomic locus is cleaved by the Cas9–sgRNA complex, the molecules responsible for the repair of the double-strand break are recruited, elevating the recombination activity specifically at the locus. Thus, the observed additional recombination was probably caused by the increase in recombination activity at the target genomic locus. Similar additional recombination was found in *P. falciparum* when nonessential genes for erythrocytic development were engineered by the CRISPR/Cas9 system using a plasmid that contained the donor template (Supplementary Fig. 4)^9,19^. Conversely, when an essential gene for erythrocytic development was mutated in our previous study, such recombination was never found despite using a circular plasmid carrying the donor template. However, the additional recombination might be overlooked in this case, because the transgenic parasites in which this recombination occurred died due to the functional disruption of the essential gene (Supplementary Fig. 4). Thus, whenever a circular plasmid is used for delivering the donor template, an additional recombination will occur between the integrated donor template and another copy of the plasmid as the donor template, resulting in failure or reduced efficiency of the genetic modification. Therefore, this is a serious technical problem of the CRISPR/Cas9 system when using circular plasmid DNA as the donor template.

### Genetic modification by transfecting pbcas9 with a linear form of the donor template

**Fig. 3:**
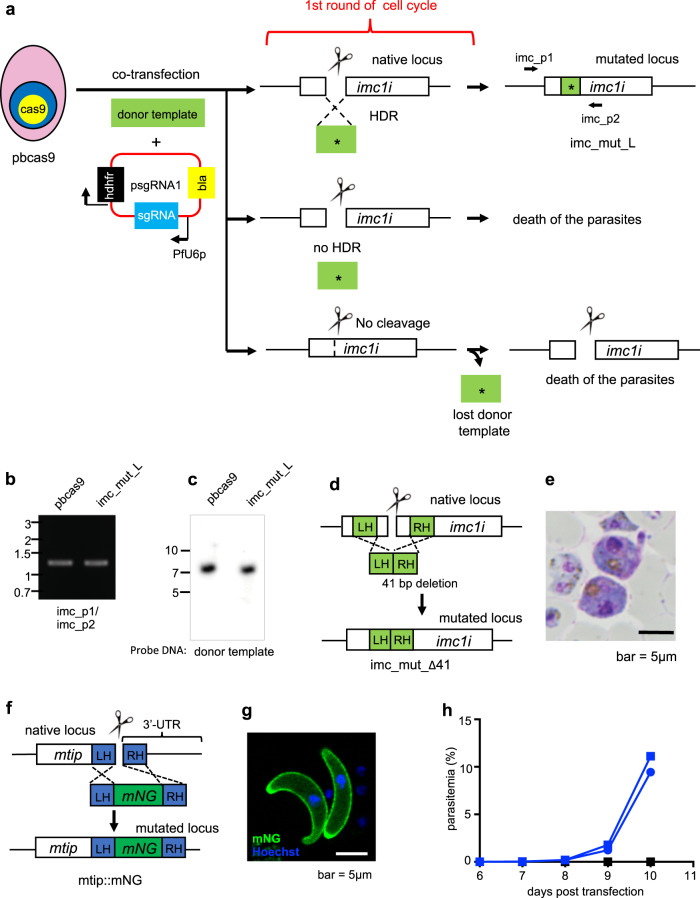
Genetic modification by the CRISPR/Cas9 system using a linear donor template. **a** The pbcas9 parasites were transfected with a linear donor template (green box) and a plasmid containing the sgRNA, resulting in a mutation of the *imc1i* gene. Top: HDR possibly occurred between the linear donor template with the mutation (asterisk) and the cleaved *imc1i* locus during the first round of the cell cycle after transfection. In addition, there were no unexpected single crossover recombinations in the resultant imc_mut_L. Middle: when the double-strand break at the target genomic locus was not repaired by HDR during the first round of the cell cycle after transfection, the parasite died due to the instability of chromosome. Bottom: when the target genomic locus was not cleaved during the first round of the cell cycle after transfection, the linear donor template was lost from the parasite and the daughter parasites would have thus only the plasmid containing sgRNA. These parasites eventually died during further cell cycles. **b** The genotyping PCR of imc_mut_L was performed using the sets of primers indicated at the bottom. **c** Southern hybridization analysis of imc_mut_L was carried out using the donor template as the DNA probe. The genomic DNA used for this analysis was purified from the transfected parasite population before cloning by limiting dilution. **d** A 41-bp sequence of the *imc1i* gene was deleted by transfecting pbcas9 with a linear donor template (green). **e** The imc_Δ41 parasites formed abnormal ookinetes. **f** The *mNG* gene was incorporated at the C-terminus of MTIP. **g** The mNG signal was detected in the pellicule of mtip::mNG ookinetes. **h** When pbcas9 were transfected with only the plasmid containing the sgRNA for MTIP, they died due to instability of the cleaved genome (black lines). In contrast, transgenic parasites were readily obtained using the linear donor template and the plasmid containing the sgRNA (blue lines). The uncropped images of gel or blot are shown in Supplementary Fig. 13.

To solve the technical problem of integrated circular donor DNA, we used a linear donor template DNA for subsequent genetic modifications. In addition, we anticipated that the linear donor template would increase transfection efficiency as reported in previous studies^2,9^. In the present study, we first tested whether the linear donor template introduced a nonsense mutation in the *imc1i* gene (Fig. 3a). Briefly, we separately prepared the linear form of the donor template (5 μg) and the plasmid containing the sgRNA, psgRNA1 (Supplementary Fig. 5), (5 μg) and cointroduced them into the pbcas9 parasites. Transfection experiments were independently carried out in duplicate. Transfected parasites were treated with pyrimethamine for 5 days and became visible in peripheral blood 2 days after drug withdrawal. The obtained parasite was named imc_mut_Linear, indicated as imc_mut_L, and the genomic DNA was purified from harvested parasites. The PCR analysis of imc_mut_L using primers imc_p1 and p2 showed that the region used for HDR could be amplified, unlike in the case of imc_mut_C (Figs. 2b and 3b and Supplementary Fig. 13). Furthermore, southern hybridization analysis using the donor template as the DNA probe showed a single signal at ~7.3 kb, identical to the wild-type parasites (Fig. 3c and Supplementary Fig. 13). In contrast, southern hybridization analysis using the *bla* gene as a probe did not detect any signals in the imc_mut_L. These results clearly showed that there was no integration of additional copies of donor template or plasmid into the parasite genome. Subsequent sequence analysis of the obtained fragment showed that the nonsense mutations at Tyr15 and Glu18 were successfully introduced (Supplementary Fig. 6a). The chromatogram obtained by this sequence analysis showed only mutated sequences. These results indicated that there were no residual wild-type parasites, suggesting that the genetic modification by our CRISPR/Cas9 system was achieved with high accuracy. In addition, clonal parasites obtained by limiting dilution possessed only mutated sequences. These clonal imc_mut_L could not form normal ookinetes, indicating the disruption of the function of IMC1i (Supplementary Fig. 6b). All these results demonstrated that the technical limitation of the CRISPR/Cas9 system could be solved by using a linear form of the donor template.

We examined whether the developed CRISPR/Cas9 system using pbcas9 could be used for gene deletion with high efficiency as in the case of the site-directed mutagenesis experiment. To this end, we attempted to delete a 41-bp sequence from the coding region of the *imc1i* gene (Fig. 3d). The linear donor template (5 μg) and the plasmid containing the sgRNA (5 μg) were cointroduced into the pbcas9 parasites, followed by drug treatment for 5 days. The transgenic parasites imc_Δ41 were obtained 2 days after withdrawal of the drug, and their genomic DNA was purified. Sequence analysis using purified genomic DNA showed only a 41-bp deletion in *imc1i* (Supplementary Fig. 7a), but not the wild-type imc1i sequence, supporting the high level of accuracy of our CRISPR/Cas9 system. The imc_Δ41 parasite could not form normal ookinetes (Fig. 3e), indicating the disruption of the function of IMC1i. Next, we attempted to fuse the fluorescent protein mNeonGreen (mNG) with the myosin A tail domain interacting protein (MTIP) (Fig. 3f). MTIP is the molecule responsible for motility and is specifically expressed in invasion stages, including merozoite, ookinete, and sporozoite^20^. The guide RNA was designed to cleave upstream of the MTIP termination codon. We cotransfected pbcas9 with a linear donor template (5 μg) and the plasmid containing the sgRNA (5 μg) and eventually obtained the transgenic parasite mtip::mNG 2 days after drug treatment for 5 days. The PCR-based genotyping analysis of harvested mtip::mNG detected only the mutant fragment, indicating a highly efficient mNG fusion to MTIP (Supplementary Figs. 7b and 13). The insertion of mNG was further confirmed by sequencing the amplified fragment (Supplementary Fig. 7c). The expression of the mNG-fused MTIP was observed in merozoites and ookinetes by fluorescence microscopy (Fig. 3g and Supplementary Fig. 7d). All of these results showed that our CRISPR/Cas9 system using pbcas9 and a linear donor template could be used not only for site-directed mutagenesis but also for gene deletion and fusion of protein.

The present study clearly showed that genes could be engineered by transfecting pbcas9 with a linear form of the donor template and a plasmid expressing the sgRNA with high accuracy. To avoid unexpected recombination at the target genomic locus, a linear form of the donor template must be used in our system. However, most of the transfected parasites quickly lose the donor template during cell division in erythrocytic development, where four to five rounds of nuclear division occur, because linear DNA segregates into daughter parasites with considerably low efficiency. Thus, the initial cleavage of the target locus and subsequent HDR with the linear donor template must be completed prior to parasites entering the next cell cycle (Fig. 3a, Top). Constitutive expression of Cas9 makes it possible to form the complex with the sgRNA immediately after transfection, soon followed by cleavage of the target genomic locus. This immediate cleavage prompts the subsequent HDR, allowing the completion of the genetic modification within the first cell cycle after transfection. Our results showed that transgenic parasites with genetic modifications always emerged in peripheral blood 2 days after drug treatment, suggesting that there were probably ~2.3 × 10^2^ transgenic parasites in which those two steps were completed within the first cell cycle after transfection. Therefore, we consider that constitutive expression of Cas9 is essential for efficient genetic modification using the linear donor template.

Owing to the absence of a cNHEJ pathway and the infrequency of MMEJ in *Plasmodium* parasites^13^, the genomic cleavage induced by the Cas9–sgRNA complex is predominantly repaired by HDR using the donor template. However, if repair by HDR does not occur, the parasites will die due to the instability of the chromosome. Thus, if there was no HDR during the first cell cycle after transfection, the parasites would die before the next cell cycle (Fig. 3a, Middle). Moreover, if the target genomic locus was not cleaved during the first cell cycle, the parasites lost the donor template during schizogony, the daughter parasites would thus have only the sgRNA-expressing plasmid. These parasites would eventually die during subsequent cell cycles (Fig. 3a, Bottom). This was evidenced when pbcas9 parasites were transfected with only the plasmid containing the sgRNA for *mtip*; transgenic parasites were never obtained (Fig. 3h). Thus, only parasites in which HDR occurred between the donor template and the cleaved site were able to survive, resulting in high accuracy of genetic modification by the present CRISPR/Cas9 system.

### Absence of off-target mutations in the transgenic pbcas9 parasites

The Cas9 nuclease–sgRNA complex can generally bind to double-stranded DNA, even if there are three to five base pair mismatches in the PAM-distal region of the sgRNA sequence. Thus, in addition to the target site, off-target sites could possibly be cleaved by the Cas9–sgRNA complex. This cleavage at off-target sites is repaired in eukaryotic cells, such as human and mouse cells, by the cNHEJ pathway with a small deletion or insertion, causing off-target mutations. However, if off-target sites are cleaved in *Plasmodium* parasites by the Cas9, the double-strand breaks might not be repaired due to the lack of the cNHEJ pathway, resulting in their elimination from the transgenic parasite population (Supplementary Fig. 8). To test this hypothesis, we conducted a genome-wide sequence analysis of the clonal imc_mut_L parasites. Whole-genome sequencing of the *imc1i*-mutated parasite was carried out at a depth of 35.9× coverage, followed by calling the mutations based on a comparison with the reference genome of *P. berghe*i ANKA in the database (Supplementary Data 3). In total, 408 mutations were called and then compared with the 407 variants called in pbcas9 using a similar procedure (Supplementary Data 3). Most mutations, except for the introduced nonsense mutations in *imc1i*, were commonly found between both the imc_mut_L and the parental pbcas9 parasites (Supplementary Data 3), indicating that they were inherited from parental *P. berghei* ANKA and were not thus mutations caused by off-target cleavages. Although 83 different mutations between the pbcas9 and *imc1i* mutant parasites were found, most of them were located within the subtelomeric regions and highly A/T rich intergenic regions (Supplementary Data 4). These mutations were possibly false positives because mapping errors frequently occurred in those regions due to their low sequence complexity and incomplete sequence information of subtelomeric regions. Therefore, off-target mutations were not generated in the course of genetic modifications.

### Double genetic modification using the Cas9-expressing parasites and two sgRNAs

**Fig. 4:**
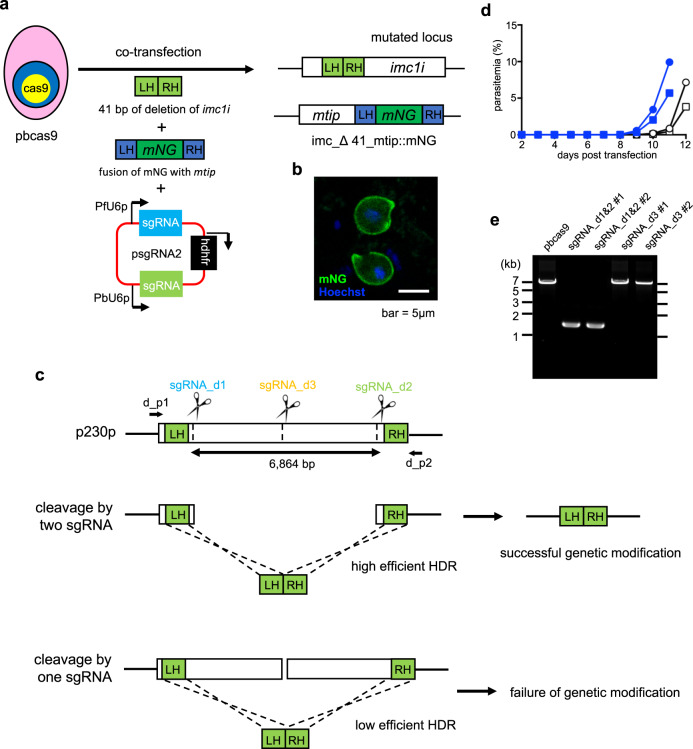
Genetic engineering based on the developed CRISPR/Cas9 system using two sgRNAs. **a** The pbcas9 was transfected with two linear donor templates and psgRNA2, resulting in the imc_Δ41_mtip::mNG. **b** The imc_Δ41_mtip::mNG parasites expressed mNG-fused MTIP in the pellicule and formed abnormal ookinetes due to the disruption of *imc1i*. **c** The genomic locus of p230p was cleaved by Cas9 and two sgRNAs, sgRNA_d1 and _d2, followed by integrating a linear donor template (green) through HDR. In contrast, when p230p was cleaved using sgRNA_d3, genetic modification failed. **d** Transgenic parasites generated using two sgRNAs emerged 8 days after transfection (blue lines). However, the parasites that were electroporated with one sgRNA became visible after 10 days (black lines). **e** The genotyping PCR of obtained parasites in **d** was performed using the primers d_p1 and d_p2 (**c**). The uncropped gel image of are shown in Supplementary Fig. 13.

During genetic modifications using our CRISPR/Cas9 system, the target locus was considered to be immediately cleaved after transfection and quickly repaired by HDR using the linear donor template. Furthermore, only transgenic parasites in which the double-strand break was repaired by HDR could survive due to the lack of the cNHEJ pathway. These observations suggested that even if two target genomic loci on different chromosomes were cleaved, only transgenic parasites in which both cleavage sites were repaired would be able to survive, resulting in simultaneous multiple modifications. To test this hypothesis, we generated a plasmid with two different sgRNAs, which were transcribed from the U6 promoters of *P. falciparum* and *P. berghei*. We then attempted the multiple genetic modifications, which were a 41 bp of deletion in the imc1i gene on chromosome 7 and a fusion of mNG with MTIP on chromosome 14 (Fig. 4a). We cotransfected pbcas9 with two different donor templates and the plasmid, psgRNA2 (Supplementary Fig. 9), containing two sgRNAs, followed by drug treatment for 5 days. The transgenic parasites emerged in blood 2 days after terminating drug treatment and were then harvested. The PCR analysis of the harvested parasites clearly showed deletion of *imc1i* and the fusion of mNG to MTIP (Supplementary Figs. 10 and 13). We did not detect wild-type parasites in this analysis, suggesting most of the emerged parasites have both mutations. Then, the mutant parasite was named imc_Δ41_mtip::mNG. These results were supported by the sequence analysis of the *imc1i* and *mtip* genomic loci of the clonal imc_Δ41_mtip::mNG parasites obtained by limiting dilution. The clonal parasites formed ookinetes with abnormal shape and expressed mNG on their surface (Fig. 4b). All these results demonstrated that two genetic modifications on different chromosomes could be carried out simultaneously by our developed CRISPR/Cas9 system.

We next utilized our CRISPR/Cas9 system using two sgRNAs to delete a kilo-bp-scale region of the genomic sequence. The gene encoding 6-cysteine protein, p230p, of which the coding region was 6864 bp, was selected as a target gene. The p230p is involved in male gamete fertility and is dispensable for blood-stage development^21^. Two guide RNAs, sgRNA_d1 and _d2, were designed near the N- and C-termini of p230p, and the donor template was generated by fusing its N- and C-terminal regions (Fig. 4c). The pbcas9 parasites were cotransfected with this linear donor template and the plasmid containing the two sgRNAs. Approximately 6 kb of the coding region of p230p was completely deleted in transgenic parasites obtained after drug screening (Figs. 4d, e and Supplementary Fig. 13). When sgRNA_d3 was used alone (Fig. 4c), the parasites emerged in peripheral blood 4 days after drug withdrawal, but PCR analysis showed that there was no expected deletion in them (Figs. 4d, e and Supplementary Fig. 13). Subsequent sequence analysis of harvested parasites detected only the wild-type genotype, suggesting that these emerged wild-type parasites would have survived due to insufficient drug treatment. The results showed that the efficiency of HDR was dependent on the distance between the cleavage site and the regions used for HDR (Supplementary Fig. 11). After the target sites were cleaved by Cas9, the DNA sequence around the 5′ end on either strand was trimmed, generating 3′ overhangs. These overhangs invade the donor template, initiating HDR. When two sgRNAs were used, the overhangs could invade efficiently, because the cleaved sites were near the regions used for HDR. In contrast, in the case when one sgRNA was used, this process was inefficient due to the distance between the cleavage site and the regions used for HDR. Thus, to achieve efficient deletion, the site targeted by the sgRNA and the regions used for HDR should be as close to each other as possible.

### Large-scale genome editing using a donor template DNA with telomere sequences

**Fig. 5:**
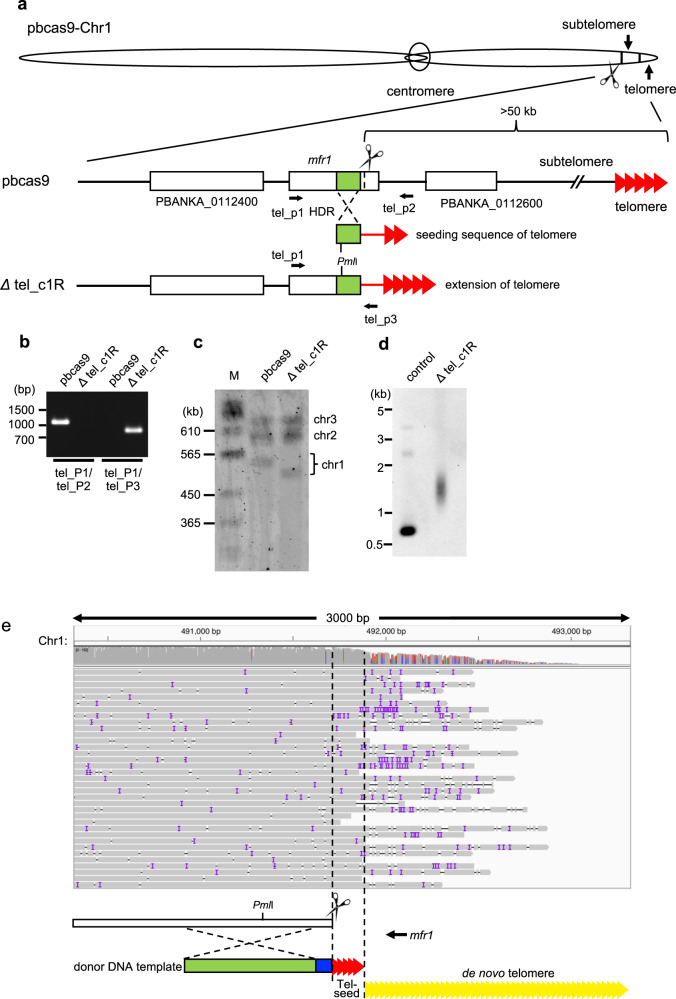
Large-scale genome editing using a donor template DNA with telomere sequences. **a** The subtelomeric region, which was estimated to be ~70 kb, was removed by the CRISPR/Cas9 system using a linear donor template with the telomeric seed sequence, resulting in the Δtel_c1R. *mfr1* was selected as the targeted genomic locus. **b** The genotyping PCR of Δtel_c1R was performed using the sets of primers indicated at the bottom. **c** The CHEF analysis of Δtel_c1R showed its small chromosome 1 compared with that of pbcas9. **d** Southern hybridization analysis of Δtel_c1R detected the broad signal caused by extension of the telomere. **e** NGS analysis of chromosome 1 shows extension of the de novo telomere sequence. Extension of the de novo telomere was confirmed by mapping long reads to the end of the truncated chromosome 1. In this analysis, we used the truncated chromosome 1 with an extra 1.6 kbp of the telomere sequence at the downstream of telomere seeding sequence. Truncated *mfr1* is represented by the open box. The donor template used for HDR and the spacer sequence are indicated in green and blue, respectively. The seeding sequences of the telomere and the de novo telomere are shown in red and yellow, respectively. The uncropped images of gel or blot are shown in Supplementary Fig. 13.

We further applied our developed CRISPR/Cas9 system for large-scale genome editing by using a telomere sequence. Since telomeres protect the ends of linear chromosomes, de novo chromosome ends are created by adding telomeric sequences to one end of the donor template, and this would allow large-scale genome editing to occur (Fig. 5a). To prove this, we attempted to remove the subtelomeric region where multiple genes responsible for immune evasion are located^22^. To avoid undesirable effects on normal parasite development in the erythrocytic stage, we selected the putative major facilitator superfamily-related protein *mfr1* gene (PBANKA_0112500) on chromosome 1 as the target for cleavage according to the phenotypic database deposited in PlasmoDB^3^. Because all genes that are located between the telomere end and the *mfr1* gene are dispensable for erythrocytic stage development, the removal of this subtelomeric region would not affect parasite development in erythrocytes (http://plasmoDB.org). The pbcas9 parasites were cotransfected with a linear donor template containing the telomere sequence and the plasmid containing the sgRNA (Fig. 5a). The genotypic analysis of the obtained transgenic parasite Δtel_c1R showed the integration of the donor template with the telomere at the cleaved site (Fig. 5b and Supplementary Fig. 13). Contour-clamped homogenous electric field (CHEF) electrophoresis of Δtel_c1R showed smaller chromosome 1 compared with the wild-type chromosome (Fig. 5c and Supplementary Fig. 13). In addition, southern hybridization analysis using the sequence close to the additional telomere as a DNA probe detected a broad signal in transgenic parasites, suggesting the extension of telomere length (Fig. 5d and Supplementary Fig. 13). Subsequent NGS sequencing analysis using nanopore technology demonstrated extension of the telomere sequence at cleaved chromosome 1 (Fig. 5e). The average size of the extended telomere sequence was ~1.2 kb, which is similar to the length of the original telomere in *P. berghei* (Supplementary Fig. 12a)^23^. In addition, NGS analysis using short reads showed that chromosome 1 was accurately cleaved at the *mfr1* locus (Supplementary Fig. 12b), and further that there was no unexpected recombination and rearrangement between the de novo and original telomere ends. Thus, these results show that large-scale chromosomal deletions can be achieved by our CRISPR/Cas9 system using a donor template and telomere sequence.

## Discussion

In conclusion, the constitutive expression of Cas9 and the usage of a linear donor template are essential for the presently developed CRISPR/Cas9 system in parasites. If either these elements are missing, robust and highly accurate genetic modification cannot be achieved. The transgenic parasites generated by this method are selectable marker free and can be reused for further genetic modifications. Since our CRISPR/Cas9 system was applicable for a wide range of genetic modifications, including mutation, deletion, tagging with fluorescence protein, and large-scale chromosome editing, transgenic parasites with multiple modifications can be generated, contributing to the study of parasites in the postgenomic era. Following this success in rodent malaria parasites, we will attempt to generate *P. falciparum* with the Cas9 nuclease gene integrated into its genome. Our approach will be further applied for all *Plasmodium* species and will thus become the standard method for their genetic modification.

## Methods

### Animal ethics

All mouse experiments were performed following the Guidelines for the Care and Use of Laboratory Animals and were approved by the Animal Experiment Committee of the Tokyo Medical and Dental University. Female ddY mice (5–7 weeks old) were used for the infection of *P. berghei* except where otherwise noted.

### Generation of *P. berghei* constitutively expressing Cas9

Cas9 from *Streptococcus pyogenes* was used in this study. This Cas9 nuclease does not cleave double-stranded DNA in the absence of sgRNA^24^ and is thus suitable for generating the parasite which express it constitutively. The pcssu-Cas9-hy plasmid (Supplementary Fig. 1a) was constructed as described in plasmid construction and used for the generation of the Cas9-constitutively expressing parasite line, i.e., pbcas9. The integration of the Cas9 cassette was performed using the pcssu-Cas9-hy plasmid by the same procedure as described previously^25^. Briefly, 10 μg of the pcssu-Cas9-hy was digested by *Kpn*I/*Not*I, and the linearized fragment was then introduced into purified schizonts (1 × 10^7^) of *P. berghei* ANKA using the parasite nucleofector II kit and the Nucleofector II device with the U-033 program (Lonza). Because the linearized fragment encoded not only the expression cassette of the Cas9 nuclease but also that of the fused gene of the positive selection marker, i.e., *hdhfr*, and the negative selection marker, i.e., *yfcu*, the selectable markers were integrated along with the Cas9 expression cassette in the genome of parasites. Transfected parasites were injected into mice intravenously immediately after electroporation. Treatment with pyrimethamine was initiated 30 h post infection and continued for 5 days, followed by withdrawal of the drug. To examine the integration of the cassettes of the Cas9 nuclease gene and the *hdhfr–yfcu* gene, PCR-based genotyping was performed using specific primer sets (Supplementary Data 1) and genomic DNA purified from obtained parasites. After confirming the integration of the cassettes, the transgenic parasite clone, i.e., pbcas9-hy parasites, was isolated by limiting dilution. To remove the *hdhfr–yfcu* gene from the parasite clone, *yfcu*-based negative selection using 5-FC was performed as described previously with some modifications^26^. Briefly, mice were infected with the pbcas9-hy parasites by intraperitoneal injection and were then treated with 5-FC for 5 days by administrating drug in water after the parasitemia reached 0.01–0.05%. We further selected the parasite clone in which only the Cas9 cassette was integrated by limiting dilution. To confirm the integration of the Cas9 expression cassette and the removal of the *hdhfr–yfcu* gene, genotyping of the obtained parasite clones was performed by PCR using the primer set cas_p4 and cas_p6 (Supplementary Data 1). The resultant drug marker-free parasite was named pbcas9 parasite and used for all CRISPR-Cas9 experiments in the present study. The sequences of oligonucleotides used for genotyping PCR are listed in Supplementary Data 1.

### Construction of sgRNA-expressing plasmid and preparation of donor template DNA

The 19-bp guide RNA sequence was designed upstream of PAM (NGG), and pairs of complementary oligonucleotides were synthesized for each target site. To limit the possibility of off-target effects, the guide RNA sequence was analyzed using the ChopChop program (https://chopchop.cbu.uib.no). Since the U6 promoter of eukaryotes including *Plasmodium* parasites requires a guanosine nucleotide to initiate transcription, guanosine was added at the 5′-end of the oligonucleotide that encoded the sense sequence. In addition, the oligonucleotides were designed to generate overhangs for cloning into *Bsm*BI- or *Bsa*I-digested sgRNA-expression plasmids, i.e., the psgRNA1 and pgRNA2 plasmids. The annealed oligonucleotides were cloned into plasmids that were used for genome editing in the pbcas9 parasite. All oligonucleotides used for generating sgRNAs are listed in Supplementary Data 1.

The donor template DNA for HDR was generated by a two-step PCR approach, except in the case of large-genome editing. To generate the donor template DNA for disrupting the gene function by site-directed mutagenesis, two DNA fragments that encoded partial sequences of the target gene, i.e.*, imc1i*, were amplified using *P. berghei* genomic DNA and two sets of primers with nonsense mutation as the initial step. One set of primers was imc_p3 and imc_Ter-R and another was imc_Ter-F and imc_p4. The primers were designed to generate PCR products with 30-bp overlap and to introduce point mutations in the PAM sequence to prevent recleavage by the Cas9–sgRNA complex. In the second step, the two PCR products were fused by PCR using the set of primers imc_p3 and imc_p4, resulting in the linear donor template DNA. To produce the donor template for deleting the gene sequence, two DNA fragments that encoded two distal regions of the target gene, i.e.*, imc1i*, were amplified by PCR. These products overlapped each other and were fused by subsequent PCR. For the construction of the fragment used in the fusion of the gene with mNG, two partial sequences that encoded the C-terminal region of *mtip* and its 3′-UTR were amplified by PCR, followed by flanking the mNG with these products by subsequent PCR. The mNG-coding sequence was chemically synthesized to be codon-optimized for *Plasmodium berghei* (GenScript: https://www.genscript.com/). Codon optimization was done automatically by the GenScript software. The termination codon was eliminated from the obtained donor template DNA.

### Gene knockout and gene knock-in by the CRISPR/Cas9 system with pbcas9 parasites

Purified schizonts (10^6^–10^7^) were transfected with 5 µg of the sgRNA-expression plasmid and 5 µg of donor DNA. For the double genetic modification, two 5 µg of donor DNA were used for the transfection in addition to the plasmid encoding the sgRNAs. The transfected parasites were intravenously injected into mice, and the mice were treated with pyrimethamine for 5 days, followed by withdrawal of drug. Parasites will lose the plasmid carrying the sgRNA quickly in the absence of selectable pressure; 80% of transfected parasites will lose the plasmid within the first 4 days after drug withdrawal^27^. After confirming the emergence of parasites in the peripheral blood, PCR-based genotyping was performed with infected blood and specific primers. Transgenic parasite clones were obtained by the limiting dilution method, as described previously^2^.

### Removal of a subtelomeric genomic region from pbcas9 parasites

The *mfr1* (PBANKA_0112500) gene was used for the removal of a subtelomeric region from chromosome 1. All genes that are located from this gene to the telomere end on chromosome 1 are dispensable for the parasite development in erythrocytes. A partial fragment of *mfr1* was amplified and cloned into the *Sac*II/*Xho*I site adjacent to the telomere sequence on the plasmid. The telomere sequence consisted of tandem repeats of CCCT(A/G)AA, and its length was 215 bp. The sequences of oligonucleotides used in the plasmid construction are included in Supplementary Data 1. The resulting plasmids were digested by *Sac*II/*Pme*I to excise the DNA fragment containing the partial *mfr1* and telomere sequences. The purified schizonts (1 × 10^7^) were transfected with 25 µg of the digested plasmid and 10 µg of the plasmid containing sgRNA specific for *mfr1*. The transfected parasites were intravenously injected into mice, and the mice were treated with pyrimethamine for 4 days. The transgenic parasites with truncated chromosome 1 were cloned by limiting dilution and were then subjected to CHEF analysis.

### Off-target analysis related to genome editing using pbcas9 parasites

Genomic DNA samples used for whole-genome sequencing were purified from pbcas9 parasites and *imc1i*- mutant parasites with point mutations by a standard phenol/chloroform method^28^. The obtained genomic DNA was further purified using the Nucleospin gDNA Clean-up kit (Macherey-Nagel). One hundred nanograms of each genomic DNA sample was sheared to an average size of 600 bp with Covaris S220 (Covaris). The DNA library was prepared using the KAPA Hyper Prep Kit (KAPA Biosystems) and TruSeq HT adapters (Illumina) according to the manufacturer’s instructions. Whole-genome sequencing was performed on the Illumina MiSeq platform (Illumina) with 251 bp paired-end sequencing.

The acquired Illumina sequencing reads were filtered using Trimmomatic (version 0.38, http://www.usadellab.org/cms/?page=trimmomatic) to remove low-quality reads. The filtered reads were mapped to the *P. berghei* ANKA reference (PlasmoDB, version 35) using the BWA-MEM mapping algorithm (version 0.7.17, http://bio-bwa.sourceforge.net) with the default setting. Variant calling was performed using HaplotypeCaller of GATK (version 3.8, https://software.broadinstitute.org/gatk) to detect single-nucleotide polymorphisms (SNPs) and insertions and/or deletions (indels). Comparisons of variant calls of the parental line, i.e., the pbcas9 parasite, and the mutant line, i.e., the imc_mut_L, were carried out with GenotypeGVCFs of GATK. Then, SNPs and indels were selected with standard filtering parameters. The variants that were called uniquely in the mutant line were confirmed on mapping data using the genome browser IGV (http://software.broadinstitute.org/software/igv/home) to remove false-positive variants. The whole-genome sequencing data of *P. berghei* ANKA independently performed by another research group (accession number: ERR3060803) was analyzed by the same procedures and used for confirming commonly found SNPs and indels in exons. Variants that were called in results from both pbcas9 and ERR3060803 were considered probable sequencing errors in the reference genomic sequence. All 42 probable sequencing errors are listed in Supplementary Data 2 (marked by #). The subtelomeric regions were defined as described in Supplementary Data 5.

### Sequence analysis of telomere-deleted parasites

Whole-genome sequencing of the Δtel_c1R parasites was performed to confirm the absence of subtelomeric regions. The DNA library was prepared using the same method as described above and analyzed using the Illumina HiSeq 2500 platform (Illumina) with 251 bp paired-end sequencing. The acquired Illumina sequencing reads were mapped to the *P. berghei* ANKA reference (PlasmoDB, version 35) using the BWA-MEM mapping algorithm with the default setting. The uniquely mapping result was visualized by IGV.

Long-read sequencing of the genome of the Δtel_c1R parasites was performed to determine the extension of the de novo telomere end. 2.5 µg of genomic DNA was used for library preparation using a Ligation Sequencing Kit according to the manufacturers’ protocol. Sequencing runs were performed using a MinION instrument (Oxford Nanopore Technologies). One hundred and fifty-seven reads with 81 bp plasmid-derived spacer sequences and a *Pml*I site in *mfr1* were selected as reads containing the de novo telomere end using local BLAST analysis. The selected reads containing the de novo telomere end were mapped to virtual chromosome 1 with extended telomere (~1600 bp) using the BWA-MEM mapping algorithm with the default setting, then the mapping result was visualized by IGV. In addition, the 157 long-read sequences were used to determine the length from the *Pml*I site to the read end of each individual read.

### Plasmid construction

To introduce the expression cassette of Cas9 nuclease in the genome of *P. berghei*, the pcssu-Cas9-hy plasmid was constructed. The pcssu-Cas9-hy contained not only the Cas9 expression cassette but also the *hdhfr–yfcu* expression cassette as the positive/negative selection marker. The human dihydrofolate reductase gene, i.e., *hdhfr* confers pyrimethamine resistance to the parasites and was used as a positive selectable marker. The *yfcu* gene is a fusion gene of yeast cytosine deaminase and uridyl-phosphoribosyltransferase. The parasite that expresses the *yfcu* gene is killed by 5-FC, and the yfcu can thus be used as a negative selection marker^4^. The Cas9 cassette and the *hdhfr–yfcu* cassette contained the 3′UTR of *hsp70*, which was used for the termination of transcription of both genes. To generate the *hdhfr–yfcu* fusion gene cassette, *hdhfr* with the *ef1α* promoter was amplified by PCR using pSK-1^25^ as a template and the primers Pef1α-F and hdhfr-R, while *yfcu* with the 3′UTR of *hsp70* was amplified from pf-gRNA^15^ with the primers yfcu-F and hsp3UTR-R. These two amplified fragments were fused by PCR. In the fused fragment, the termination codon of *hdhfr* was eliminated to generate the fusion gene. The resulting *hdhfr–yfcu* cassette was cloned into the *Sal*I/*Bam*HI sites of pSK-1, resulting in the pSK-1-hy plasmid. The Cas9 expression cassette was excised by *Nhe*I/*Sal*I from the pfCas9 plasmid^15^ and cloned into the pSK-1-hy plasmid, resulting in the pSK-1-Cas9-hy plasmid. To integrate the Cas9 and *hdhf-yfcu* cassettes into the genomic locus of the rRNA C-type subunit (*cssu*) on chromosome 5, two partial sequences, HDR1 and 2, of the *cssu* were amplified and cloned into the *Kpn*I/*Xho*I site and the *Bam*HI/*Not*I site of pSK-1-Cas9-hy, resulting in the pcssu-Cas9-hy plasmid (Supplementary Fig. 1a).

The plasmid containing the sgRNA-expression cassette of (psgRNA1) was previously generated and used for genome editing in this study. The psgRNA plasmid contains the sgRNA-expression cassette under control of the *P. falciparum* U6 RNA promoter (PF3D7_1341100) and the positive selection marker *hdhfr*. Two *Bsm*BI restriction sites were designed between the sequences of the U6 promoter and the DNA sequence of the sgRNA scaffold and used for cloning the DNA-targeting sequence of the sgRNA. The map of psgRNA1 was represented in Supplementary Fig. 5.

To generate the plasmids for the expression of two different sgRNAs, the *P. berghei* U6 promoter (780 bp, PBANKA_1354380) was used in addition to the PfU6 promoter. The *P. berghei* U6 promoter was amplified with the genomic DNA of the *P. berghei* ANKA strain and the primers PbU6-F and PbU6-R. In addition, the same sgRNA scaffold sequence in psgRNA1 was amplified using the specific primers sgRNA-scaffold-F and sgRNA-scaffold-R. These two PCR fragments were fused by PCR. The resulting PbU6-sgRNA cassette contained two restriction sites for *Bsa*I between the PbU6 promoter and sgRNA scaffold sequences and was used for cloning the DNA-targeting sequence of the second sgRNA. The *Bsa*I site of the beta-lactamase gene of psgRNA1 was eliminated by site-directed mutagenesis with the primers ΔBsaI-F and ΔBsaI-R prior to the cloning of the PbU6-sgRNA cassette. Subsequently, the PbU6-sgRNA cassette was cloned into the *Not*I/*Bam*HI sites of the mutated psgRNA1 plasmid lacking the *Bsa*I site, resulting in the psgRNA2 plasmid. The map of psgRNA2 was represented in Supplementary Fig. 9.

The plasmid containing the sgRNA targeting *imc1i* and the *imc1i* donor template (psgRNA_donor) was used for the first genetic modification trial. The linear donor DNA template for *imc1i*-mutagenesis was cloned into the *Bam*HI/*Not*I sites of the psgRNA1 plasmid in which the sgRNA targeting *imc1i* was cloned using the primer set imc_p3_BamHI and imc_p4_NotI, resulting in the psgRNA_donor plasmid.

To generate the donor template with a telomeric seed sequence, a partial sequence of *mfr1* was amplified from the genomic DNA of *P. berghei* ANKA using the primer set mfr1-F and mfr1-R and cloned into the *Sac*II/*Xho*I sites of the plasmid PACv1^28^. The resultant plasmid was named pch1_tel. All oligonucleotide sequences of PCR primers and the sgRNA are listed in Supplementary Data 1.

### Evaluation of the development of pbcas9 in the course of life cycle

Asexual blood-stage replication was evaluated by monitoring parasitemia in infected mice. Frozen stocks of pbcas9 were injected into naive female BALB/c mice (6 weeks old). After parasitemia reached ~1.0%, 1000 iRBCs were transferred intravenously into new naive mice. Progress of parasitemia was examined every 24 h using a Giemsa-stained thin blood smear. Averages of parasitemia between pbcas9 and wild-type parasites were evaluated using a *t*-test. Ookinete formation was evaluated as previously described with some modifications^29^. Briefly, mice were pretreated with an intraperitoneal injection of 0.2 mL phenylhydrazine (6 mg/mL in PBS) 3 days prior to parasite infection to stimulate reticulocyte formation. At 5 days post infection, infected blood was collected from mice, and white blood cells were removed using cellulose powder D (Advantec, #49020040). Leukapheresis blood was diluted tenfold with ookinete culture medium (RPMI1640 containing 100 µM xanthurenic acid, 50 mg/L hypoxanthine, 25 mM HEPES, 24 mM NaHCO_3_, 50 U/mL penicillin, 50 µg/mL streptomycin, and 20% FCS, adjusted to pH 7.5). Diluted blood samples were incubated at 20 °C for 22–24 h. Infectivity of pbcas9 mosquitoes was examined as previously described^30^. After confirming the number of exflagellated parasites in the infected blood (>50 per 10^5^ RBC), mice were subjected to bites of *Anopheles stephensi* mosquitoes (5–7 days old). The engorged mosquitoes were selected and maintained at 20 °C. Numbers of oocysts in the midgut and sporozoites in the salivary glands were counted on 14 and 24 days, respectively, after feeding. To evaluate the infectivity of pbcas9 sporozoites to liver cells, 30,000 salivary gland sporozoites were injected intravenously into Wistar rats (3 weeks old). The prepatent period was determined by microscopically monitoring the emergence of parasites in the peripheral blood every 24 h.

### Immunocytochemistry

A thin smear of schizont-rich culture of *P. berghei* was used for immunocytochemistry. Immunocytochemistry was carried out as previously described with some modifications^29^. Briefly, parasites on the slides were fixed in ice-cold acetone for 10 min and then blocked in 3% BSA-PBS for 30 min. The fixed parasites were incubated with primary antibodies overnight at 4 °C, washed, and then incubated with secondary antibodies for 60 min at room temperature. The slides were mounted with ProLong Diamond Antifade Mountant with DAPI (Thermo Fisher Scientific) and analyzed under a standard fluorescence microscope (Olympus). Mouse anti-FLAG M2 antibody (1:1000; Sigma, F1804-200UG) was used for the detection of FLAG-tagged Cas9. Alexa488-conjugated goat anti-mouse IgG (H + L) (1:200, Jackson #A-11029) was used as a secondary antibody.

### Fluorescence imaging

Fluorescence of mNG was analyzed after staining the nuclei with Hoechst 33342 (1 µg/mL). Fluorescent images of the ookinetes were acquired with the SP8 confocal microscope (Leica Microsystems). Image processing was performed using LAS AF (Leica Microsystems) and ImageJ (NIH).

### Western blotting

Infected red blood cells were purified by removing leukocytes by passing through a column of cellulose powder D and then lysed with red blood cell lysis buffer (150 mM NH4Cl, 10 mM NaHCO3, and 1 mM EDTA). After red blood cell lysis, the parasites were recovered by centrifugation and dissolved in 1x SDS-loading buffer containing 5% 2-mercaptoethanol, followed by boiling for 5 min. Western blotting was performed as described previously^31^. In brief, parasite proteins (1 × 10^7^ parasites per lane) were separated by SDS-PAGE and transferred to a PVDF membrane. The blotted membrane was blocked in TBST containing 4% skimmed milk, incubated for 90 min with primary antibodies in the same buffer, washed, and then incubated for 60 min with horseradish peroxidase-conjugated secondary antibody. Mouse anti-FLAG M2 antibody (1:1000; Sigma, F1804-200UG) was used for the detection of the FLAG-tagged Cas9 nuclease. Mouse anti-PbHSP70 antiserum (1:200; gift from Dr. Hirai^32^) was used as the internal control. HRP-conjugated goat anti-mouse IgG (H + L) (1:10000, Jackson 115-035-146) was used as a secondary antibody. The HRP signals were visualized using Immobilon Western Chemiluminescent HRP Substrate (Millipore) and detected with ChemiDoc MP (Bio-Rad).

### Estimation of initial number of transgenic parasites in which the genetic modification was completed within the first cell cycle after transfection

The initial number of mtip::mNG transgenic parasites, in which the genetic modification was completed within the first cell cycle after transfection, was estimated. The transfected parasites were treated with pyrimethamine for 5 days. The number (*N*) of transfected parasites was calculated using the following equation: *N* = (*T* × *P*/100) × 1/(*M*_on_^5^ × *M*_off_^2^), where *P* is the percentage of parasitemia at 2 days after drug removal (0.19%), *T* is the total number of red blood cells in whole mouse blood (8.0 × 10^9^ RBCs per mouse), and *M* is the multiplication rate of the parasites. The multiplication rate of the parasites in the absence of drug was 7.48, which was calculated based on the growth curve in Fig. 3h, and that of the parasites with the sgRNA-expression plasmid was 4.1 in the presence of drug as previously described^28^.

### Contour-clamped homogeneous electric field electrophoresis

Contour-clamped homogeneous electric field electrophoresis was performed as described previously with some modifications^28^. Infected red blood cells were isolated after leukocyte removal using a cellulose column and were then lysed with red blood cell lysis buffer. After red blood cell lysis, the parasites were collected by centrifugation, washed with PBS, and mixed into a 2% low-melting agarose gel at a ratio of 1:1 (v/v). The resulting agarose block containing the parasites was placed in SE buffer (0.5 M EDTA at pH 8.0 and 1% sarcosyl), treated overnight with proteinase K (final concentration, 100 mg/mL), and set into a 1.5% agarose gel (PFC gel, Bio-Rad). *P. berghei* chromosomes were separated by contour-clamped homogeneous electrophoresis using a CHEF Mapper XA system (Bio-Rad) in 0.5x TBE buffer under the following conditions: initial switching time of 60 s, final switching time of 120 s, angle of 120°, voltage gradient of 6 V/cm, run time of 24 h, and temperature of 14 °C. The separated DNA was stained with ethidium bromide and imaged with ChemiDoc MP (Bio-Rad).

### Southern hybridization

Southern hybridization analysis was performed as described previously with some modifications^28^. Briefly, *P. berghei* genomic DNA extracted from blood-stage parasites (2 μg) was completely digested with the restriction enzyme *EcoR*I as indicated below. The fragments were separated on a 1.0% agarose gel and transferred to nylon membranes. To detect the *imc1i* locus, the genomic DNA was digested with *EcoR*I, and the donor DNA for HDR was used as the DNA probe. The specific sequence was amplified by PCR using psgRNA_donor as the template with the primer set imc_p3 and imc_p4. The *bla* gene was used as the DNA probe for the detection of plasmid integration. The *bla*-encoded DNA sequence was amplified from psgRNA and specific primers. To detect the de novo telomere end specifically, the genomic DNA was digested with *Pml*I, and we designed the DNA probe within the HDR region. This specific sequence was amplified by PCR using the pch1_tel plasmid as the template with the specific primers mfr1-probe-F and mfr1-probe-R. For the control experiment, the pch1_tel plasmid, used for generating the donor template DNA with telomere sequence (10 pg), was digested with *Pml*I and *Pme*I and then blotted. The PCR products were labeled with DIG and used as hybridization probes. Chemiluminescence signals were detected using ChemiDoc MP (Bio-Rad). The sequences of oligonucleotides used for the amplification of probes are listed in Supplementary Data 1.

### Statistics and reproducibility

For parasite growth in infected rodents, the values were presented as mean ± SEM from at least three biological replicates and statistically compared using the unpaired Student’s *t* test. For parasite persistence in the infected mosquitoes, the Mann–Whitney test and the unpaired Student’s *t* test were performed to compare statistical distributions of oocyst number and sporozoites number, respectively. The exact number of biological replicates was provided in individual figure legends. The statistical analyses were performed with GraphPad Prism 6.0 (GraphPad Software Inc.).

### Reporting summary

Further information on research design is available in the Nature Research Reporting Summary linked to this article.

## Supplementary information

Supplementary information

Description of Additional Supplementary Files

Supplementary Data 1

Supplementary Data 2

Supplementary Data 3

Supplementary Data 4

Supplementary Data 5

Supplementary Data 6

Reporting Summary

## Data Availability

Whole-genome sequencing data are deposited in the DDBJ database under accession number DRA009423, DRA010056, and DRA010057. Long-read sequences containing de novo telomere are deposited in the DDBJ database under accession number LC541744–LC541900 (total 157 sequences). All relevant data are available from the authors upon request. Full blots and gels are shown in Supplementary Information. Source data underlying plots shown in figures are provided in Supplementary Data 6.
