# Supplementary material for: Improvement of CRISPR/Cas9 system by transfecting Cas9-expressing Plasmodium berghei with linear donor template
Source: Commun Biol. 2020 Aug 5;3:426. doi: 10.1038/s42003-020-01138-2 (PMC7406498; doi:10.1038/s42003-020-01138-2)
Supplement: Supplementary file 1 — Supplementary information [file 42003_2020_1138_MOESM1_ESM.pdf]

Supplementary Figures

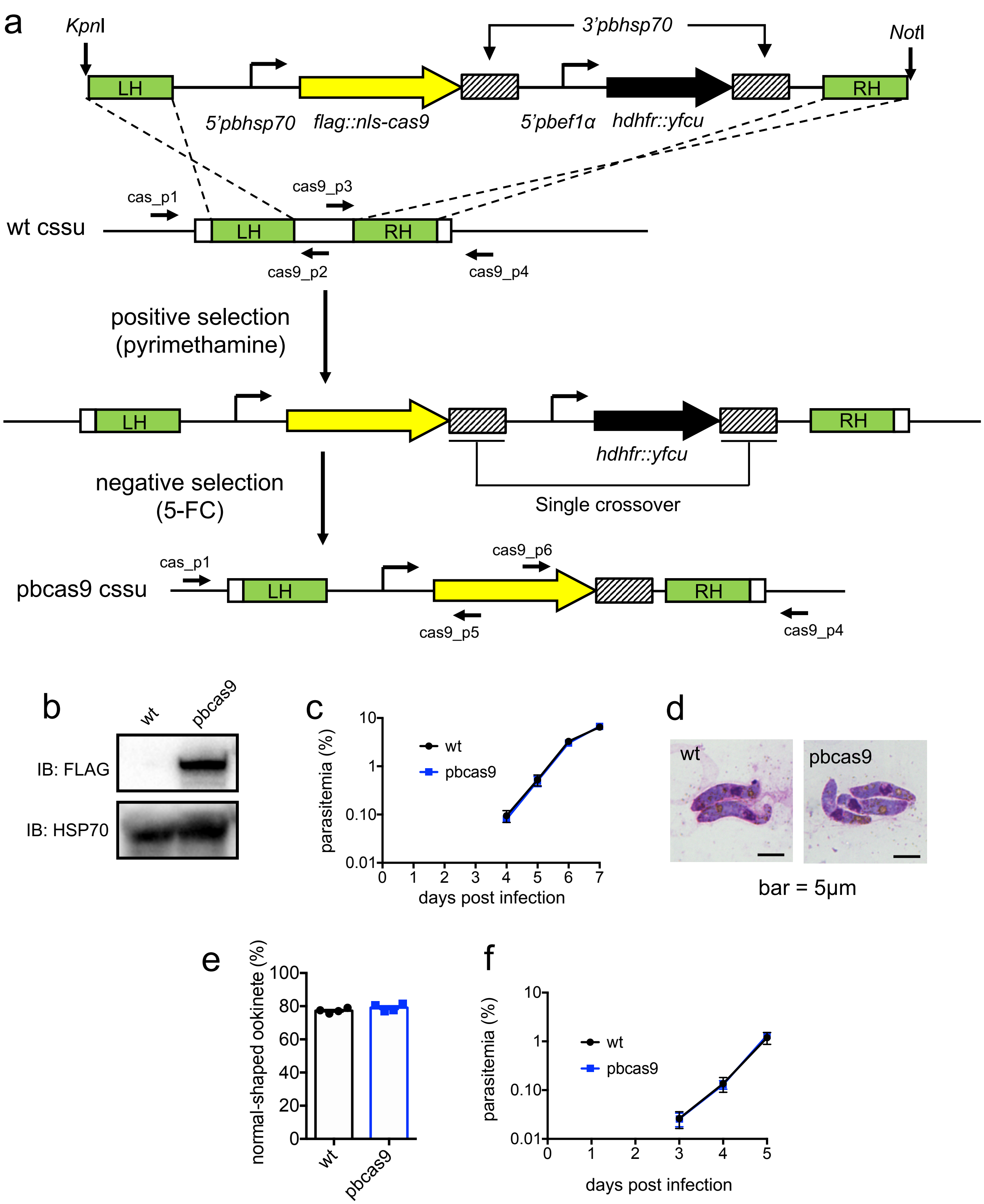

Supplementary Figure 1. Generation and characterization of the pbcas9 parasite. (a) A scheme of the generation of pbcas9 (associated with Fig. 1a). The Cas9 expression cassette (yellow) and drug-selectable marker were integrated in the cssu locus via double crossover recombination by positive selection with pyrimethamine. Subsequently, the drug-selectable marker was excised by negative selection with 5-FC via single crossover recombination. (b) The protein expression of Cas9 nuclease was detected in pbcas9 parasites by anti-FLAG antibody. Anti-hsp70 antibody was used as an internal control. The uncropped blot image of are shown in Supplementary Fig. 13. (c) The resultant progress of parasitemia in Fig 1d were log-transformed. (d and e) Ookinete shape and conversion rate were normal in pbcas9 parasites. (f) The resultant progress of parasitemia in Fig 1g were log-transformed.

PbANKA_07_v3:80,811-80,850; PBANKA_0701000

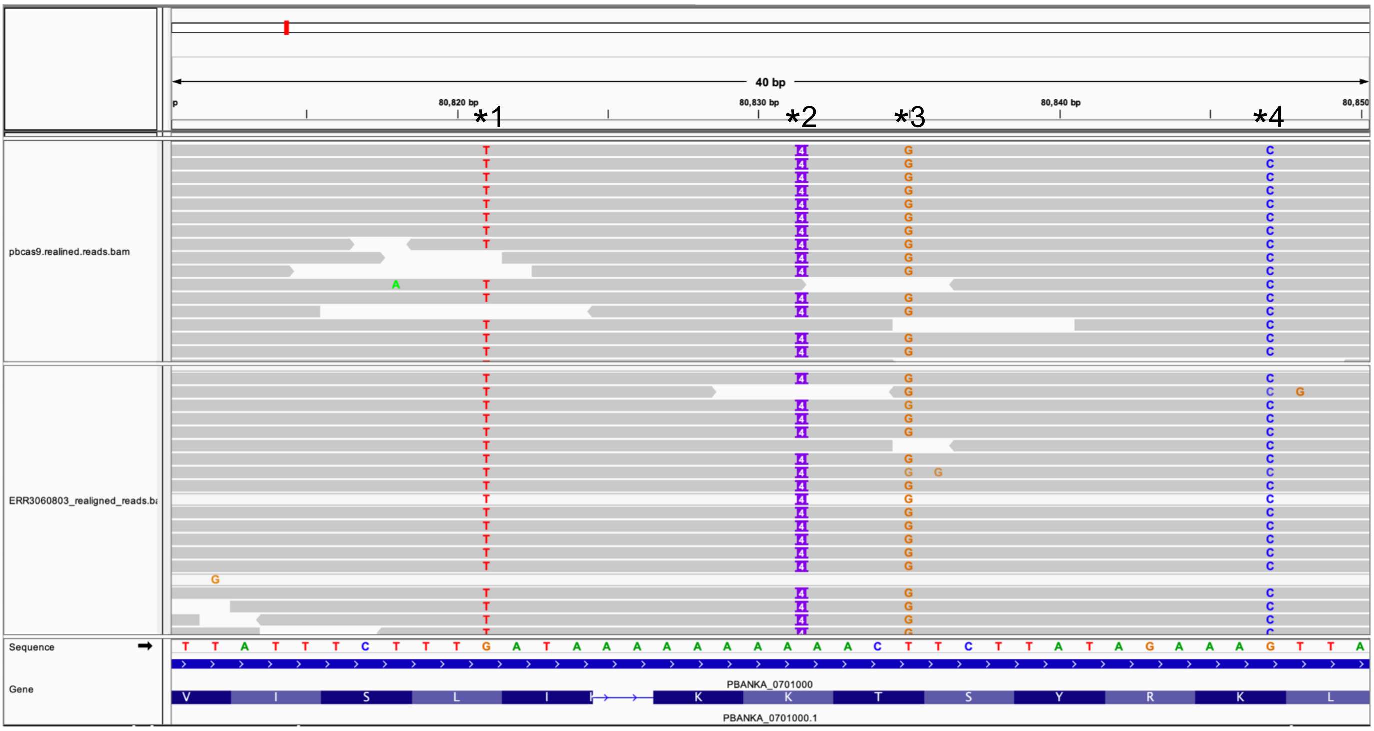

Supplementary Figure 2. Examples of sequencing errors in the reference genome. Whole-genome sequencing (WGS) data of pbcas9 and another WGS dataset (ERR3060803) identified a number of similar mutations and indels, suggesting sequencing errors in the reference genome of *P. berghei* ANKA. An IGV screenshot of the mapped reads at the PBANKA_0701000 locus; pbcas9 (top), ERR3060803 (bottom). Nucleotides marked by asterisks are the identified variants. *1 and *4 indicate nonsynonymous mutations. *2 indicates an indel. *3 indicates a synonymous mutation. These are probable sequencing errors in the reference genome. All 42 probable sequencing errors are listed in Supplementary TABLE S2 (marked with #).

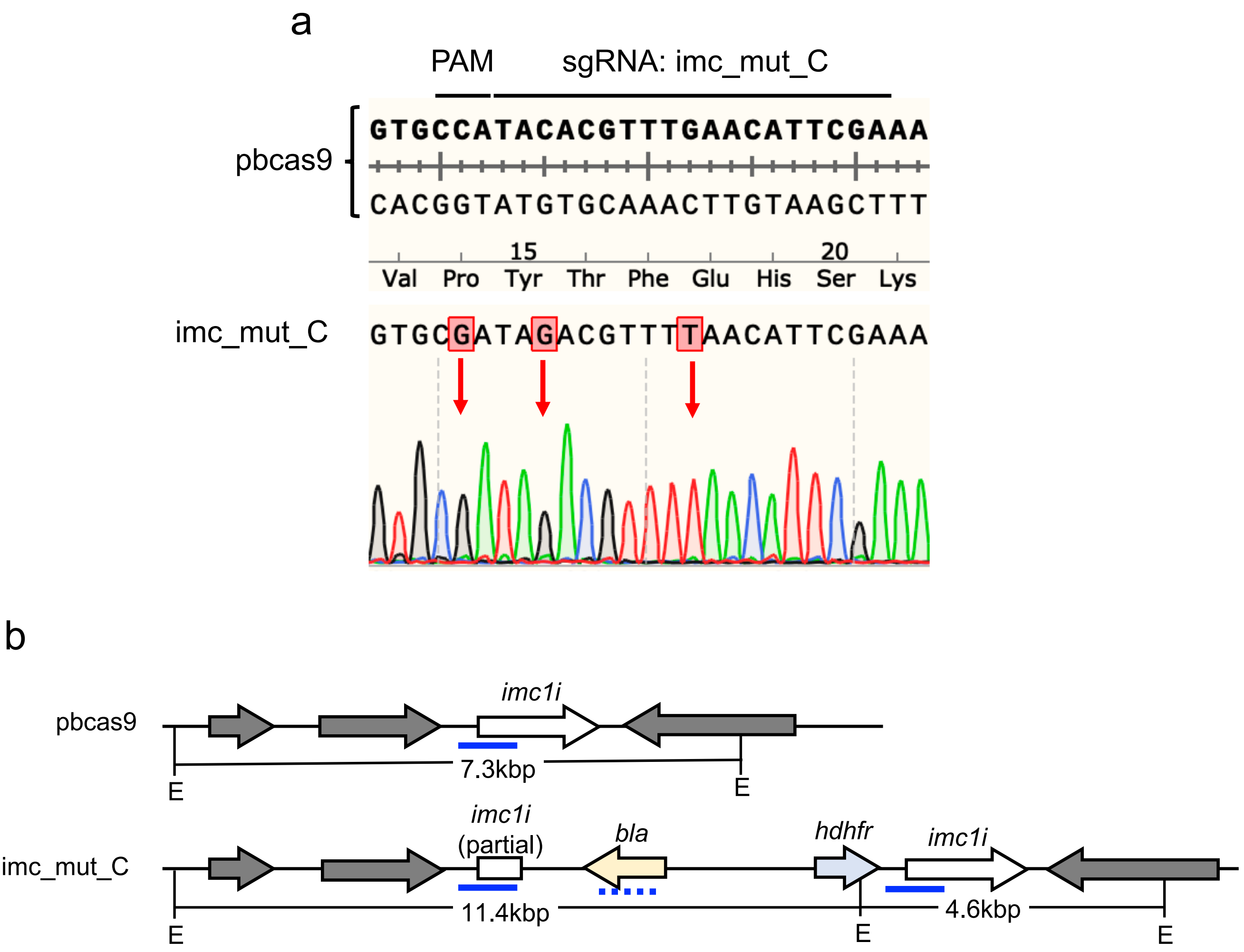

Supplementary Figure 3. Occurrence of HDR between the donor template on a plasmid and the *imc1i* locus. (a) DNA sequencing confirmed the mutations at Pro14, Tyr15 and Glu18 of the *imc1i* genomic sequence of imc_mut_C. In addition, this analysis showed that there were no wild-type sequences. (b) A schematic drawing of the *imc1i* locus of the imc_mut_C. The plasmid containing the donor template was integrated at this locus. The blue solid and dashed lines indicate the DNA probes for detecting the donor template sequence and the β-lactamase (bla) gene in Southern hybridization analysis. E indicates *Eco*RI recognition sites.

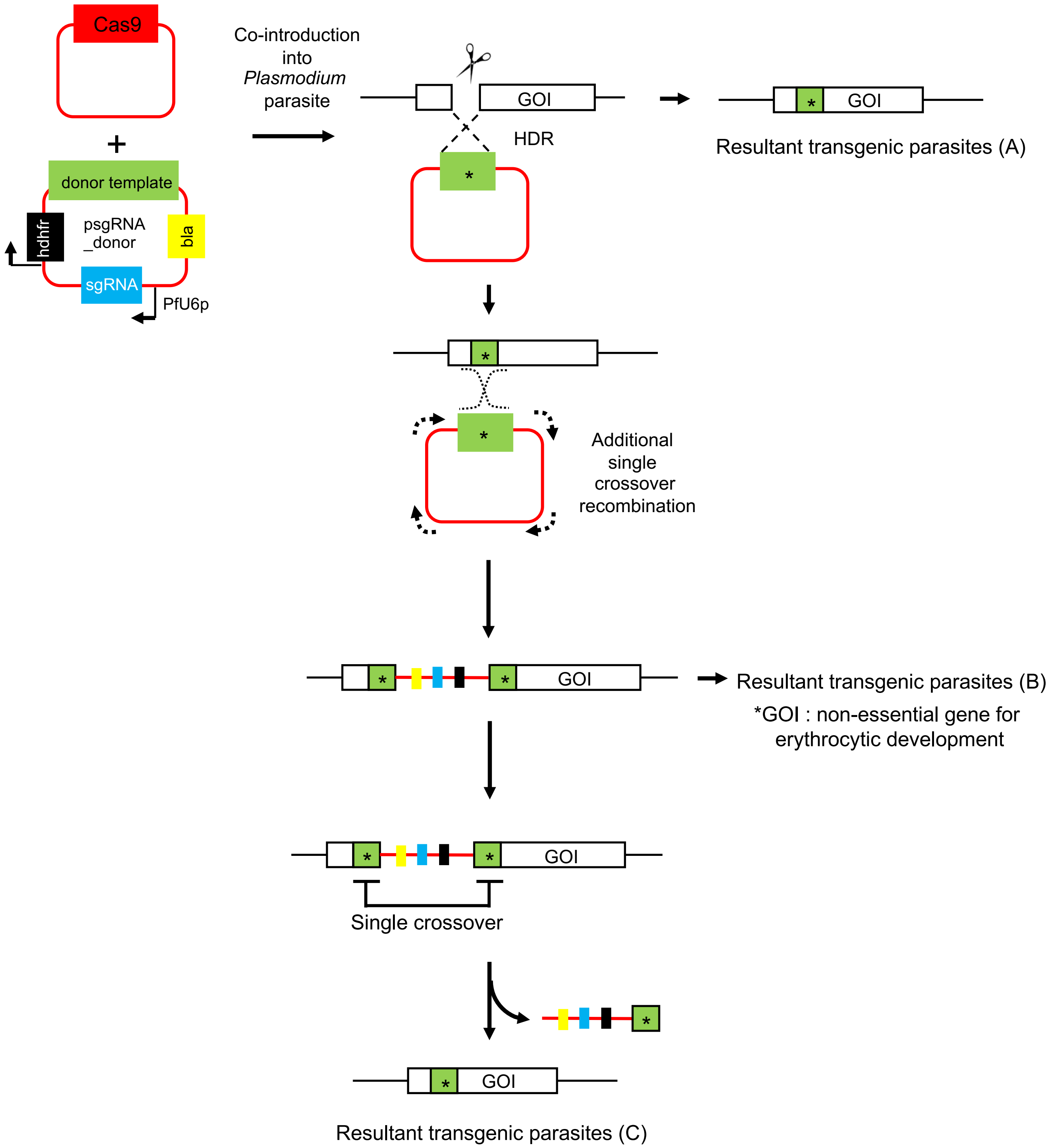

Supplementary Figure 4. The possible recombination in the parasites transfected with two plasmids having Cas9, the sgRNA, and the donor template. One plasmid encoded Cas9, and another the sgRNA and the donor template. These plasmids were cointroduced into wild-type parasites. The gene of interest (GOI) was cleaved by the Cas9-sgRNA complex, followed by HDR with the donor template on the plasmid. If there was no additional recombination, the resultant parasites had the desired mutation (A). However, if additional single crossover recombination occurred, the plasmid containing the donor template was integrated at the targeted genomic locus, resulting in an unexpected genetic modification (B). Moreover, if single crossover recombination further occurred between two donor template sequences in transgenic parasites, the integrated plasmid sequence was removed, resulting in transgenic parasites (C). The present results indicated that when *imc1i*, which is nonessential for erythrocytic development, was mutated, the majority of transgenic parasites had an integrated plasmid sequence at the targeted locus, as shown in (B).

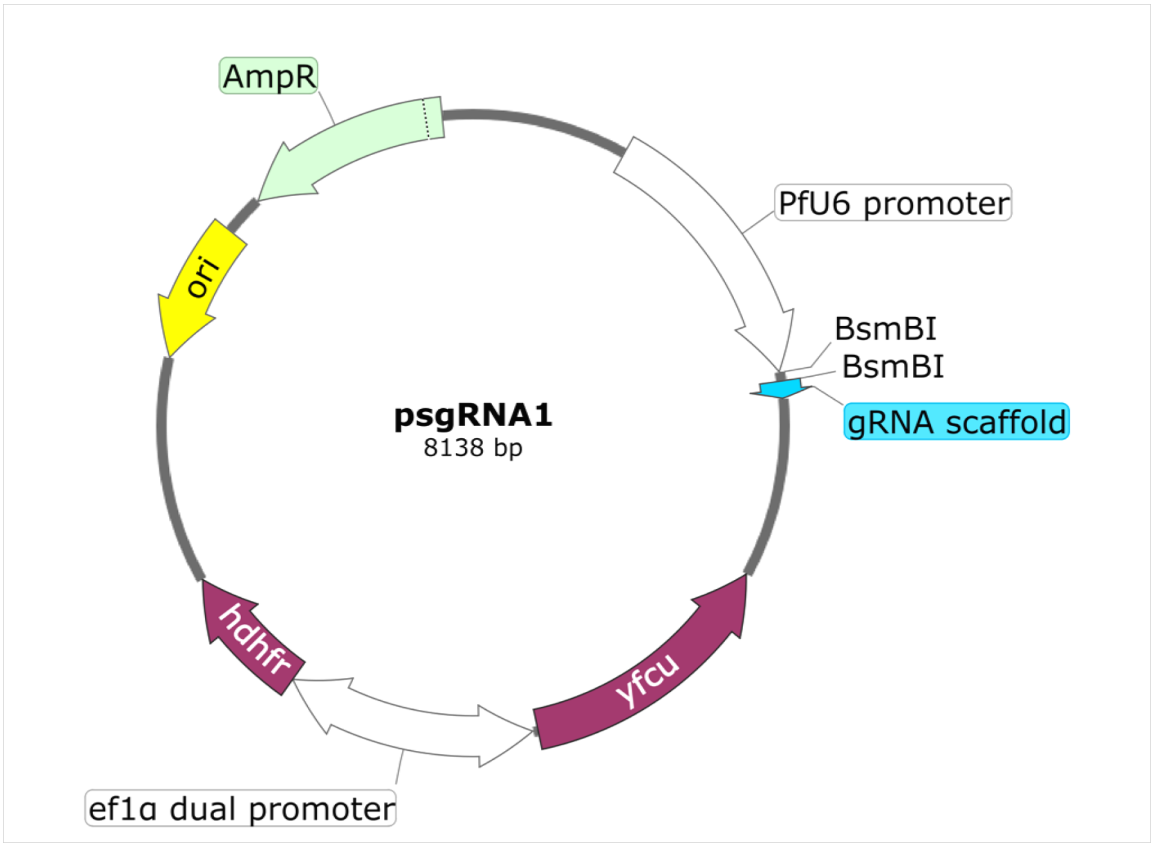

Supplementary Figure 5. Plasmid map of psgRNA1 expressing sgRNA. The sgRNA is transcribed by PfU6 promoter. The sgRNA-encoding sequence is cloned into *Bsm*BI sites located between the promoter and the scaffold sequence. The vector contains *hdhfr* as a positive-selection marker and *yfcu as* a negative selection marker. The expressions of *hdhfr* and *yfcu* are driven by ef1α dual promoter.

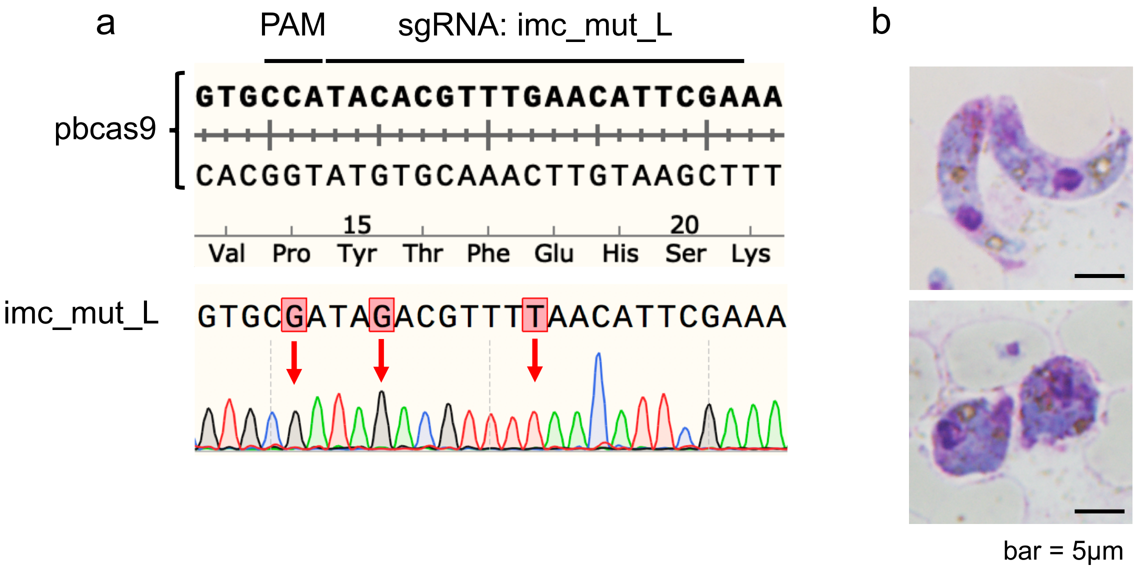

Supplementary Figure 6. Sequencing and phenotypic analysis of *imc1i*-mutated parasites. (a) DNA sequencing showed the three mutations at Pro14, Tyr15 and Glu18 in imc_mut_L. (b) The pbcas9 parasites formed normal ookinetes (upper), but imc_mut_L formed abnormal ookinetes (bottom).

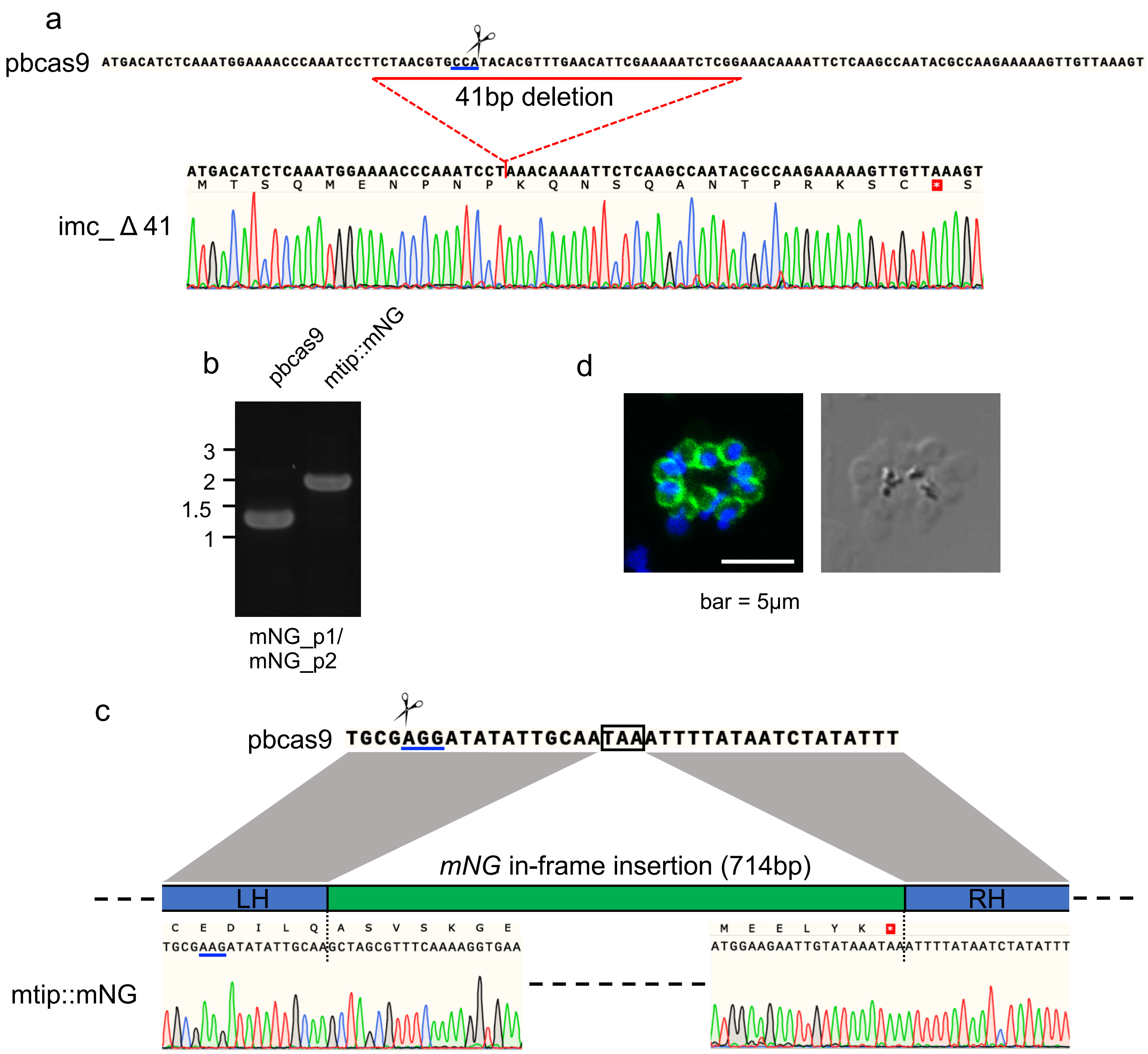

Supplementary Figure 7. Gene deletion and fusion of fluorescence protein by the CRISPR/Cas9 system using pbcas9. (a) DNA sequencing confirmed a 41-bp deletion in the *imc1i* gene in imc_Δ41 parasites. The reverse complement of targeted PAM sequence is shown with a blue line. (b) The *mNG* insertion in the *mtip* locus was confirmed by the genotyping PCR of mtip::mNG using the sets of primers indicted at the bottom. (c) DNA sequencing showed in-frame insertion of *mNG* at the 3' end of the *mtip* gene in the mtip::mNG parasite. The endogenous stop codon is shown in the open square. The targeted PAM sequence is shown with a blue line, and the shield mutation was introduced in the donor template, as indicated also by a blue line. (d) mNG expression in the pellicule of mtip::mNG merozoites.

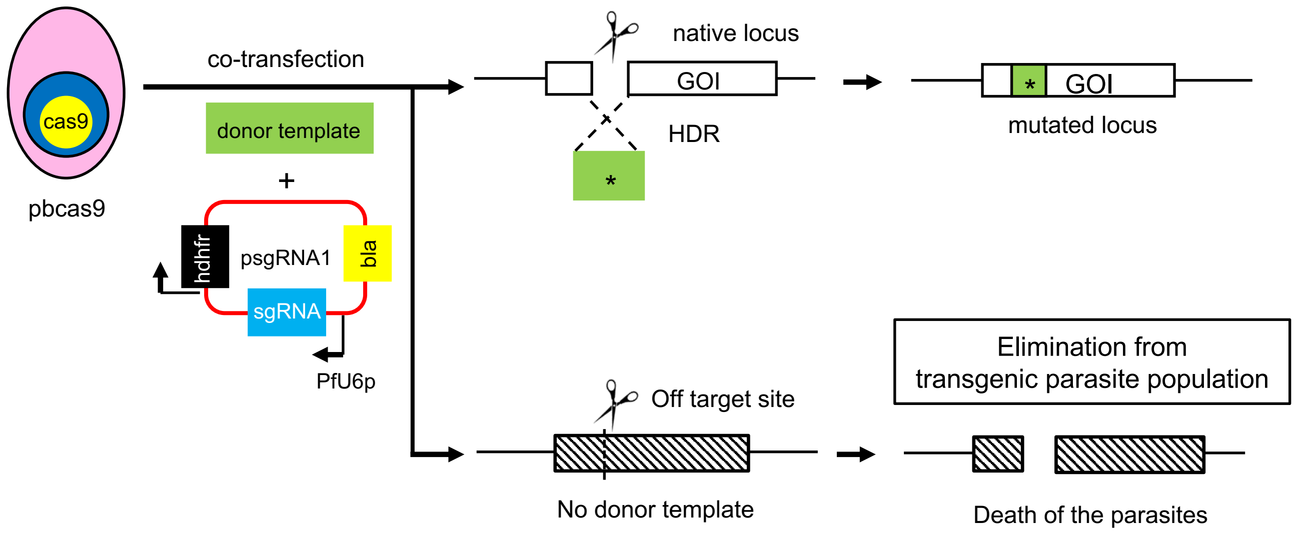

Supplementary Figure 8. Elimination of off-target mutations from transgenic parasites generated by the present CRISPR/Cas9 system. The Cas9 nuclease-sgRNA complex cleaved the target site of the gene of interest (GOI). Subsequently, HDR occurred to repair the double-strand break, and the mutated locus was generated. Off-target sites were possibly cleaved by the Cas9-sgRNA complex. These double-strand breaks were not repaired due to the lack of the cNHEJ pathway in *Plasmodium* parasites, resulting in their elimination from the transgenic parasite population.

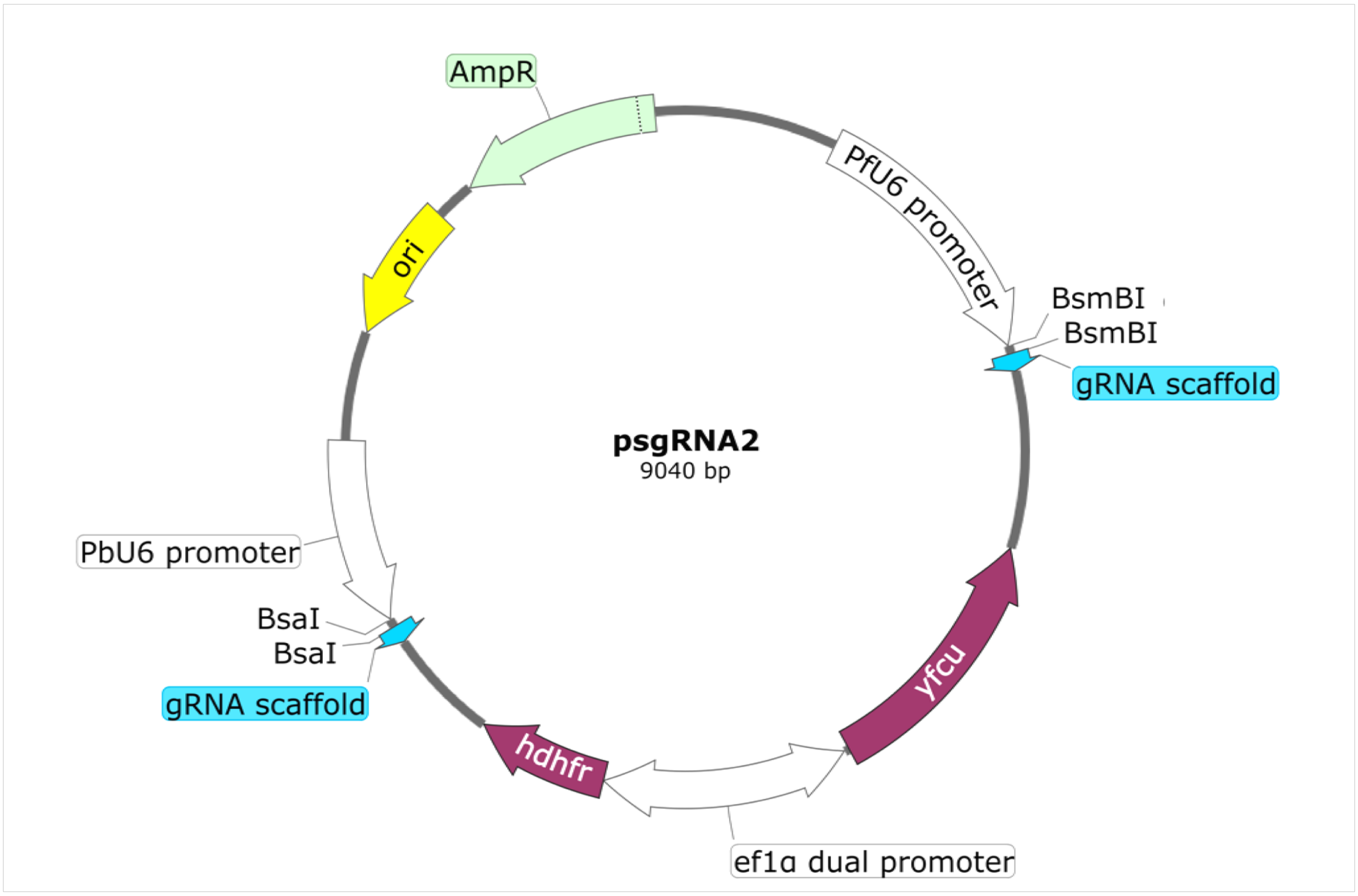

Supplementary Figure 9. Plasmid map of psgRNA2 expressing two different sgRNAs. The sgRNAs were transcribed by PfU6 promoter and PbU6 promoter, respectively. *Bsm*BI sites and *Bsa*I sites are used for the cloning of the guide RNA-encoding sequences. The vector contains *hdhfr* as a positive-selection marker and *yfcu as* a negative selection marker. The expressions of *hdhfr* and *yfcu* are driven by ef1α dual promoter.

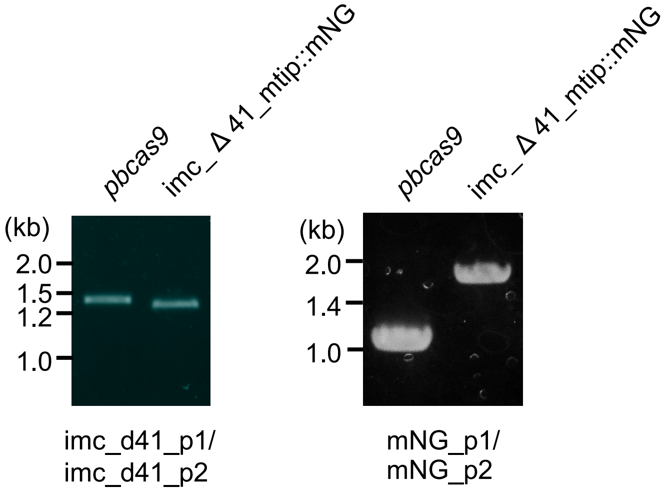

Supplementary Figure 10. PCR-based confirmation of a double genetic modification. (a) The 41-bp deletion of the *imc1i* gene was confirmed by genotyping PCR of imc_Δ41_mtip::mNG using the primers indicated at the bottom. (b) The *mNG* insertion in the *mtip* locus was also confirmed by the genotyping PCR of imc_Δ41_mtip::mNG using the primers indicted at the bottom.

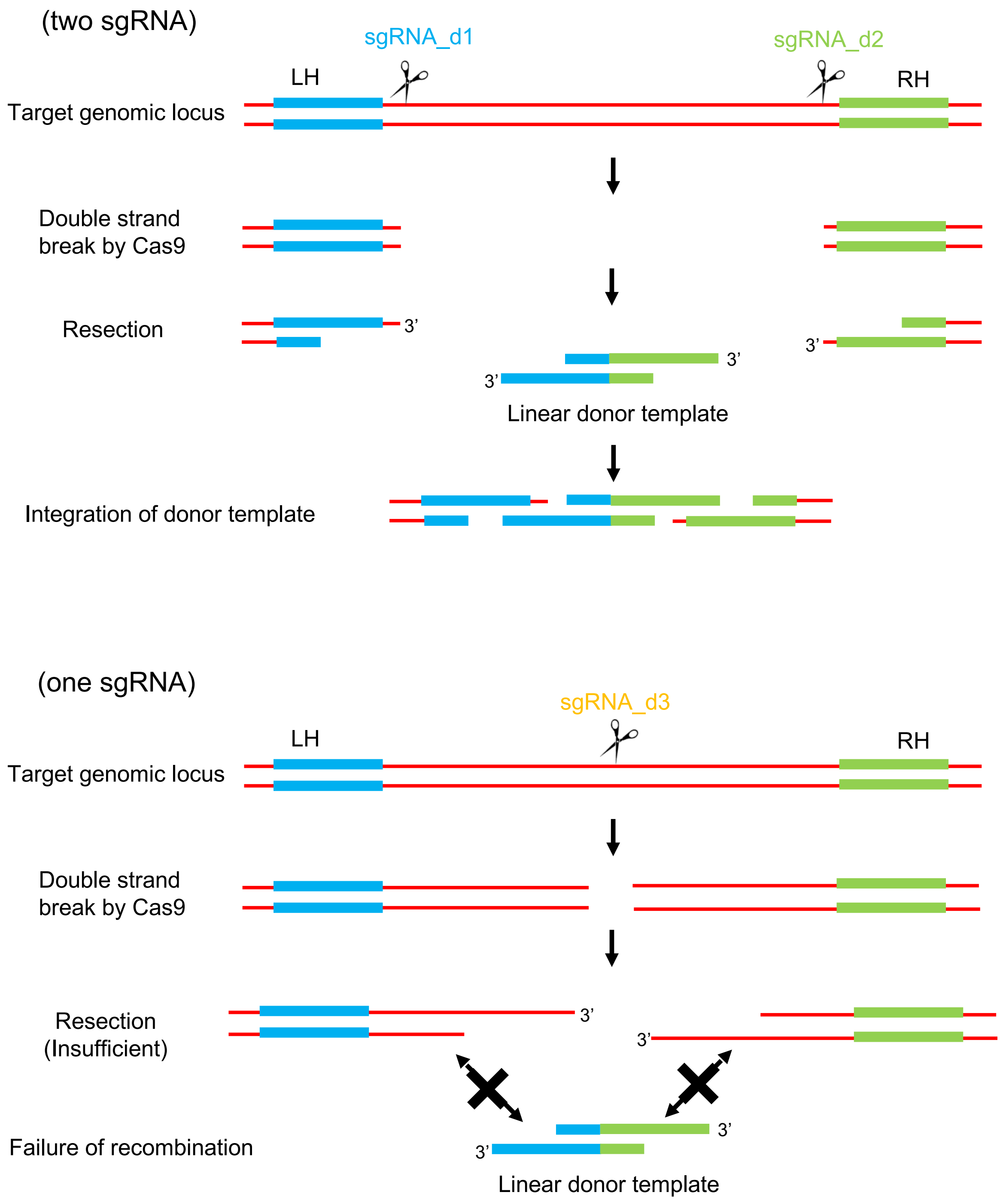

Supplementary Figure 11. Possible homology-directed recombination at the sites cleaved by Cas9 and two sgRNAs. The two sites were cleaved using sgRNA_d1 and _d2. The 5' ends of one DNA strand at both cleaved sites and the donor DNA template were resected, resulting in 3' overhangs. Using these overhangs, the donor template was integrated at the target locus. In contrast, when the target locus was cleaved by one sgRNA, *i.e.,* sgRNA_d3, the resection of the DNA sequence was too short to integrate the donor template.

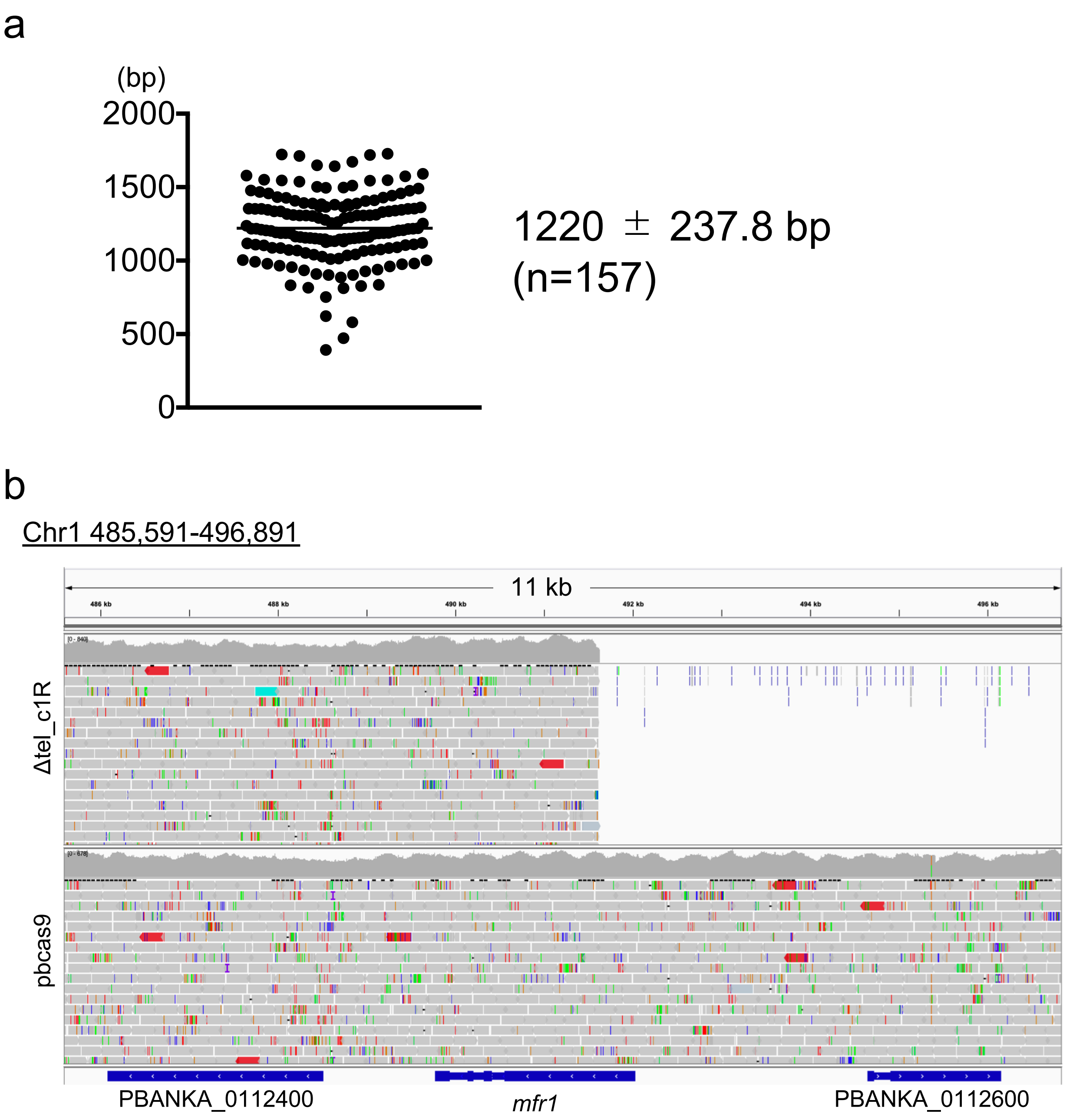

Supplementary Figure 12. Sequencing analysis of *de novo* telomere end in the telomere-cleaved mutant parasite. (a) Long read sequencing revealed the extension of de novo telomere end in Δtel_c1R. The dots represent the length from *Pml*I site to telomere end in individual long read sequences acquired by Nanopore technology and the mean is indicated by the horizontal line. The error bar indicates standard deviation (n=157). (b) Whole genome sequencing data of pbcas9 and Δtel_c1R identified that the deletion of the subtelomeric regions at the end of chromosome 1. An IGV screenshot of the mapped reads at genomic regions from PBANKA_0112400 to PBANKA_0112600. NGS reads from pbcas9 were mapped to the entire region of chromosome 1, whereas the reads from Δtel_c1R were not mapped to the regions beyond the cleaved site in *mfr1* locus.

Fig. 1b

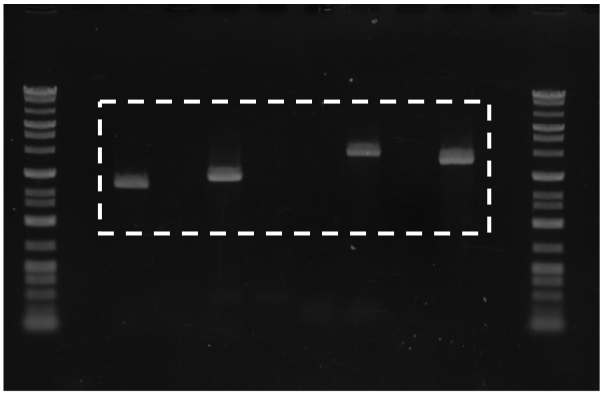

Fig. 2b

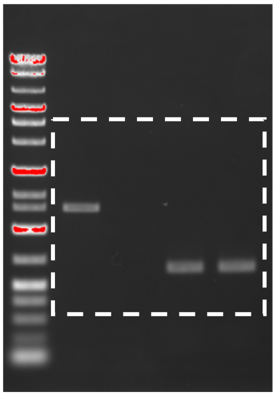

Fig. 2c

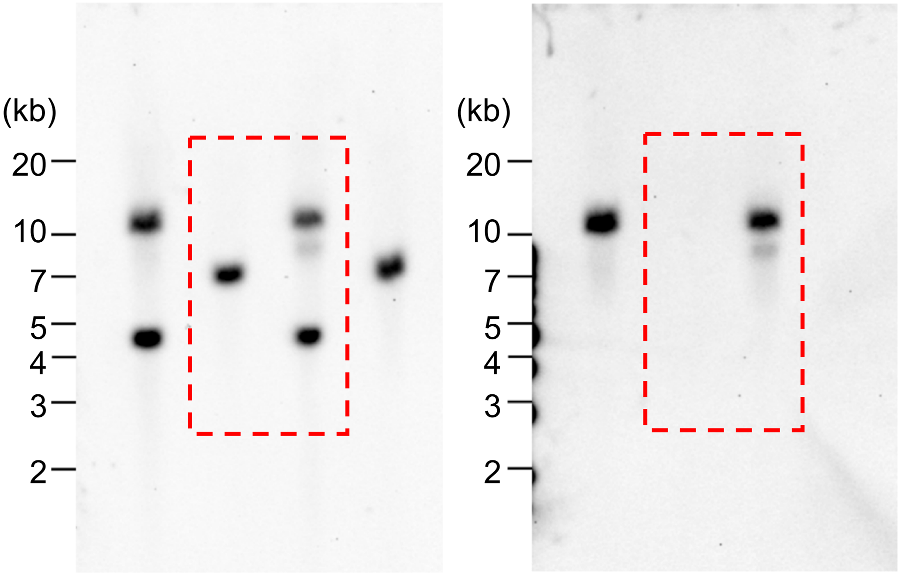

Probe: donor template

Probe:beta-lactamase

Fig. 3b

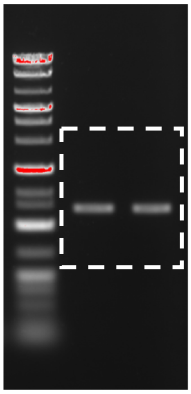

Fig. 3c

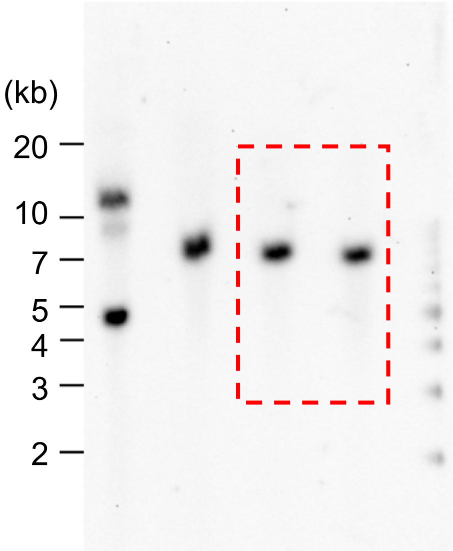

Probe: donor template

Supplementary Figure 13: Uncropped images of gel or blot. Dashed boxes indicate pictures shown in the relevant figures

Fig. 4e

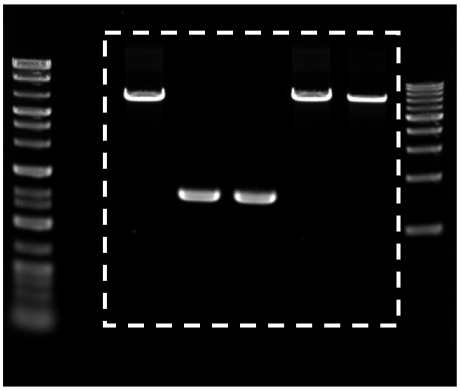

Fig. 5b

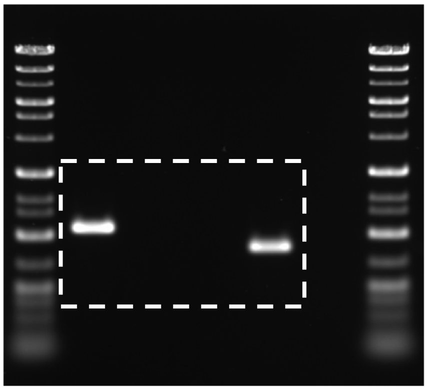

Fig. 5c

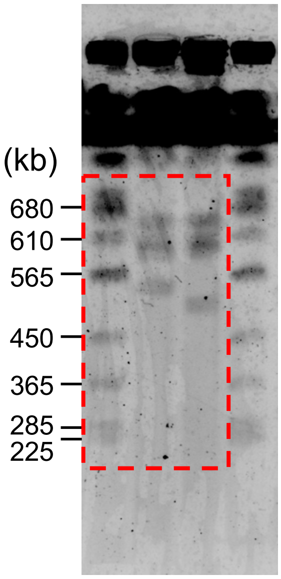

Fig. 5d

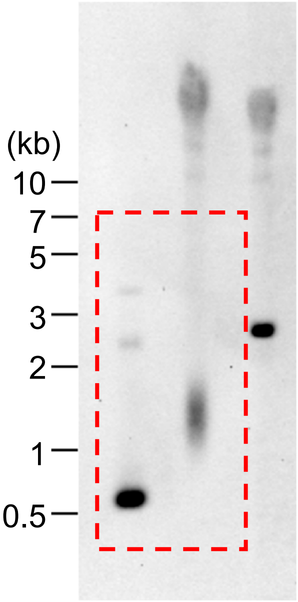

Supplementary Figure 13 (continued)

Supplementary Fig. 1b

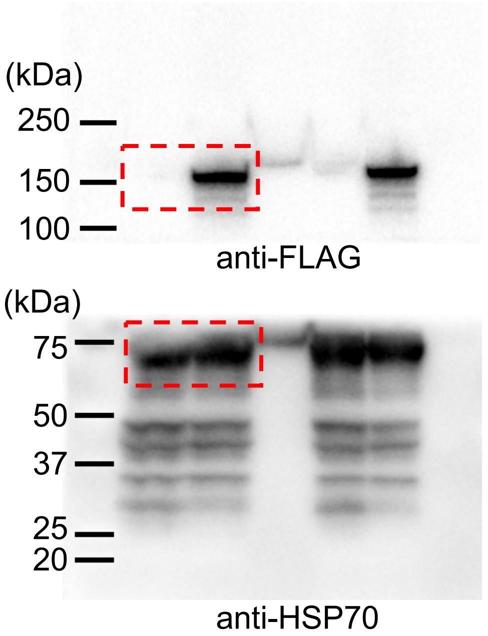

Supplementary Fig. 7b

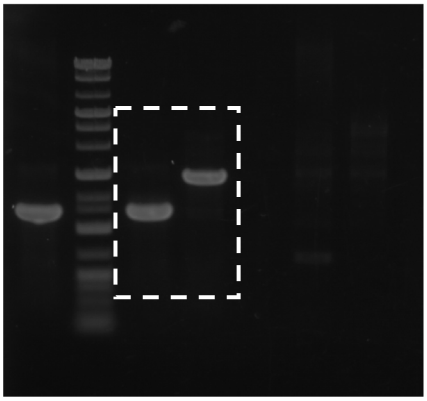

Supplementary Fig. 10

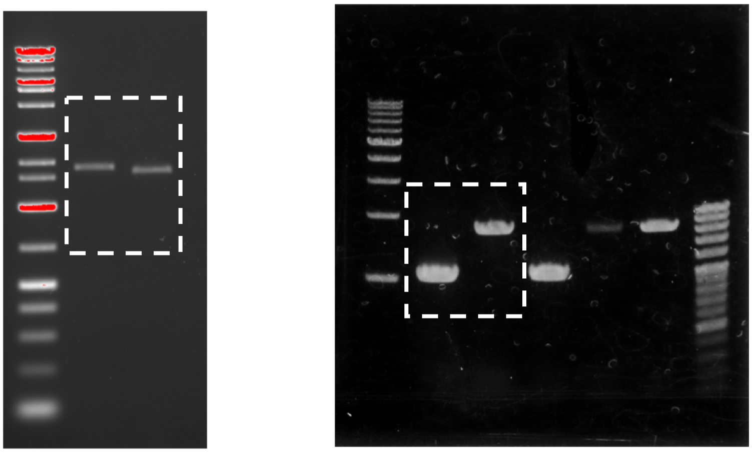

Supplementary Figure 13 (continued)
