# Supplementary material for: Improvement of CRISPR/Cas9 system by transfecting Cas9-expressing Plasmodium berghei with linear donor template
Source: Commun Biol. 2020 Aug 5;3:426. doi: 10.1038/s42003-020-01138-2 (PMC7406498; doi:10.1038/s42003-020-01138-2)
Supplement: Supplementary file 2 — Description of Additional Supplementary Files [file 42003_2020_1138_MOESM2_ESM.pdf]

Description of Additional Supplementary Files

File Name: Supplementary Data 1
Description: Oligonucleotides used in this study

File Name: Supplementary Data 2
Description: SNPs and indels in pbcas9

File Name: Supplementary Data 3
Description: SNPs and indels in imc_mut_L

File Name: Supplementary Data 4
Description: Unique SNPs and indels in pbcas9 or imc_mut_L

File Name: Supplementary Data 5
Description: The definition of subtelomeric regions

File Name: Supplementary Data 6
Description: All source data underlying the graphs presented in the main figures.
