# Supplementary material for: Improvement of CRISPR/Cas9 system by transfecting Cas9-expressing Plasmodium berghei with linear donor template
Source: Commun Biol. 2020 Aug 5;3:426. doi: 10.1038/s42003-020-01138-2 (PMC7406498; doi:10.1038/s42003-020-01138-2)
Supplement: Supplementary file 9 — Reporting Summary [file 42003_2020_1138_MOESM9_ESM.pdf]

nature research

Corresponding author(s): Shiroh Iwanaga

Last updated by author(s): Jun.25.2020

Reporting Summary

Nature Research wishes to improve the reproducibility of the work that we publish. This form provides structure for consistency and transparency in reporting. For further information on Nature Research policies, see Authors & Referees and the Editorial Policy Checklist.

Statistics

For all statistical analyses, confirm that the following items are present in the figure legend, table legend, main text, or Methods section.

n/a	Confirmed	
☐	☒	The exact sample size (*n*) for each experimental group/condition, given as a discrete number and unit of measurement
☐	☒	A statement on whether measurements were taken from distinct samples or whether the same sample was measured repeatedly
☐	☒	The statistical test(s) used AND whether they are one- or two-sided<br>*Only common tests should be described solely by name; describe more complex techniques in the Methods section.*
☐	☒	A description of all covariates tested
☒	☐	A description of any assumptions or corrections, such as tests of normality and adjustment for multiple comparisons
☐	☒	A full description of the statistical parameters including central tendency (e.g. means) or other basic estimates (e.g. regression coefficient) AND variation (e.g. standard deviation) or associated estimates of uncertainty (e.g. confidence intervals)
☐	☒	For null hypothesis testing, the test statistic (e.g. *F*, *t*, *r*) with confidence intervals, effect sizes, degrees of freedom and *P* value noted<br>*Give P values as exact values whenever suitable.*
☒	☐	For Bayesian analysis, information on the choice of priors and Markov chain Monte Carlo settings
☒	☐	For hierarchical and complex designs, identification of the appropriate level for tests and full reporting of outcomes
☒	☐	Estimates of effect sizes (e.g. Cohen's *d*, Pearson's *r*), indicating how they were calculated

Our web collection on statistics for biologists contains articles on many of the points above.

Software and code

Policy information about availability of computer code

Data collection	Whole genome sequencing: The DNA library was prepared using the KAPA Hyper Prep Kit (KAPA Biosystems) and TruSeq HT adapters (Illumina) according to the manufacturer's instructions. Whole-genome sequencing was performed on the Illumina MiSeq platform or HiSeq 2500 platform (Illumina) with 251 bp paired-end sequencing.<br>Long-read sequencing: The DNA library was prepared using a Ligation Sequencing Kit according to the manufacturers' protocol. Sequencing runs were performed using a MinION instrument (Oxford Nanopore Technologies).
Data analysis	Trimmomatic (version 0.38, http://www.usadellab.org/cms/?page=trimmomatic) was used for removal of low-quality reads.<br>The BWA-MEM algorithm (version 0.7.17, http://bio-bwa.sourceforge.net) was used for mapping of the reads.<br>HaplotypeCaller of GATK (version 3.8, https://software.broadinstitute.org/gatk) was used for variant calling.<br>Local BLAST analysis (version 2.9.0, ftp://ftp.ncbi.nlm.nih.gov/blast/executables/blast+/LATEST/) was used for selection of reads containing de novo telomere end.<br>GraphRad Prism 6.0 (GraphPad Software Inc.) was used for the statistical analyses.

For manuscripts utilizing custom algorithms or software that are central to the research but not yet described in published literature, software must be made available to editors/reviewers. We strongly encourage code deposition in a community repository (e.g. GitHub). See the Nature Research guidelines for submitting code & software for further information.

Data

Policy information about availability of data

All manuscripts must include a data availability statement. This statement should provide the following information, where applicable:

- Accession codes, unique identifiers, or web links for publicly available datasets
- A list of figures that have associated raw data
- A description of any restrictions on data availability

Whole genome sequencing data are deposited in the DDBJ database under accession number DRA009423, DRA010056 and DRA010057. Long read sequences

containing de novo telomere are deposited in the DDBJ database under accession number LC541744 - LC541900 (total 157 sequences). All relevant data are available from the authors upon request. Source data underlying plots shown in figures are provided in Supplementary Data 6.

Field-specific reporting

Please select the one below that is the best fit for your research. If you are not sure, read the appropriate sections before making your selection.

☒ Life sciences ☐ Behavioural & social sciences ☐ Ecological, evolutionary & environmental sciences

For a reference copy of the document with all sections, see nature.com/documents/nr-reporting-summary-flat.pdf

Life sciences study design

All studies must disclose on these points even when the disclosure is negative.

Sample size	Sample sizes were chosen on the basis of previous experience with the animal model employed.
Data exclusions	No data were excluded
Replication	All attempts at replication were successful. Experiments were repeated at least once and all data presented in the manuscript were successfully reproduced without exclusions.
Randomization	Mice experiments: All mice used for experiments throughout this study were female, and all within a set age range for a given experiment. Mice were allocated randomly into experimental groups prior to any experiments commencing.<br>Mosquito experiments: Fully engorged female mosquitoes were selected after blood sucking and maintained. To evaluate parasite persistence, the infected mosquitoes were randomly assigned to dissection.
Blinding	The experiments were not performed blinded. Staff resources for routine experimental blinding were not available.

Reporting for specific materials, systems and methods

We require information from authors about some types of materials, experimental systems and methods used in many studies. Here, indicate whether each material, system or method listed is relevant to your study. If you are not sure if a list item applies to your research, read the appropriate section before selecting a response.

Materials & experimental systems

n/a	Involved in the study	
☐	☒	Antibodies
☒	☐	Eukaryotic cell lines
☒	☐	Palaeontology
☐	☒	Animals and other organisms
☒	☐	Human research participants
☒	☐	Clinical data

Methods

n/a	Involved in the study	
☒	☐	ChIP-seq
☒	☐	Flow cytometry
☒	☐	MRI-based neuroimaging

Antibodies

Antibodies used	Anti-FLAG M2 (Sigma, F1804), Anti-HSP70 (gifted)
Validation	Anti-FLAG: The bulletin (https://www.sigmaaldrich.com/content/dam/sigma-aldrich/docs/Sigma/Bulletin/f1804bul.pdf)<br>Anti-HSP70: gifted from Dr. Makoto Hirai, Reference: Hino A et al., 2012 J. Biochem., (doi: 10.1093/jb/mvs058.)

Animals and other organisms

Policy information about studies involving animals; ARRIVE guidelines recommended for reporting animal research

Laboratory animals	ddY mouse (Female, 5-7 wks old), Balb/c mouse (Female, 5-7 wks old), Wistar rat (Female, 3 wks old), Anopheles stephensi (Female, 5-7 days old)
Wild animals	No wild animals were used in this study.
Field-collected samples	No field-collected samples were used in the study.
Ethics oversight	All mouse experiments were performed following the Guidelines for the Care and Use of Laboratory Animals and were approved by the Animal Experiment Committee of the Tokyo Medical and Dental University.

Note that full information on the approval of the study protocol must also be provided in the manuscript.
